# Ferrous Pyrophosphate and Mixed Divalent Pyrophosphates as Delivery Systems for Essential Minerals

**DOI:** 10.1021/acsfoodscitech.4c00050

**Published:** 2024-06-05

**Authors:** Neshat Moslehi, Michiel van Eekelen, Krassimir P. Velikov, Willem K. Kegel

**Affiliations:** † Van’t Hoff Laboratory for Physical and Colloid Chemistry, Debye Institute for Nanomaterials Science, Utrecht University, Padualaan 8, 3584 CH Utrecht, The Netherlands; ‡ Unilever Innovation Centre Wageningen, Bronland 14, 6708 WH Wageningen, The Netherlands; § Soft Condensed Matter, Debye Institute for Nanomaterials Science, Utrecht University, Princetonplein 5, 3584 CC Utrecht, The Netherlands; ∥ Institute of Physics, University of Amsterdam, Science Park 904, 1098 XH Amsterdam, The Netherlands

**Keywords:** divalent metal pyrophosphate, essential dietary minerals, ferrous pyrophosphate, iron supplement, mineral delivery systems, mixed divalent mineral salt

## Abstract

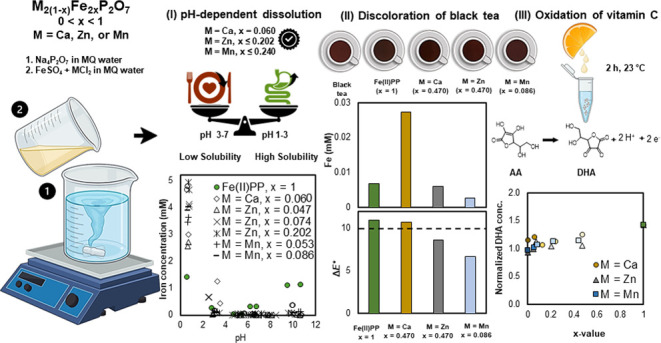

Poorly water-soluble iron-containing compounds are promising iron fortificants. However, ensuring high bioaccessibility and low reactivity of iron is challenging. We present the potential application of ferrous pyrophosphate (Fe(II)PP) and Fe(II)-containing M_2(1–*x*)_Fe_2*x*_P_2_O_7_ salts (0 < *x* < 1, M = Ca, Zn, or Mn) for delivery of iron and a second essential mineral (M). After preparation by a facile and environment-friendly coprecipitation method, the salts were investigated for their composition, pH-dependent dissolution, iron-mediated discoloration of a black tea solution, and oxidation of vitamin C. Our results suggest that these salts are possible dual-fortificants with tunable composition that compared to Fe(II)PP (i) show lower (<0.5 mM) and enhanced (to 5 mM) iron dissolution in moderate and gastric pH, respectively, (ii) exhibit less discoloration and dissolved iron in tea when *x* = 0.470 for M = Ca or Zn and *x* = 0.086 for M = Mn, and (iii) do not increase the oxidation extent of vitamin C over 48 h when *x* = 0.06, 0.086, or 0.053 for M = Ca, Zn, or Mn, respectively.

## 1 Introduction

Fortification of food products with micronutrients is a potentially successful and economical strategy to address mineral and vitamin deficiencies.^1−3^ Among dietary minerals, iron is the most deficient in the human body.^4,5^ According to World Health Organization (WHO), iron deficiency is responsible for approximately 0.8 million deaths each year.^6^ However, adding iron to foods is challenging due to the high reactivity of iron ions with phenolic compounds found in food.^7−9^ Severe changes in the organoleptic properties of the foods as well as a decrease in iron bioavailability and absorption are consequences of this reactivity.^10,11^ To tackle this issue, fortifying food products with poorly water-soluble or water-insoluble iron sources such as iron(III) pyrophosphate (Fe(III)PP) has been shown to be a beneficial and cost-effective approach.^5,12−14^ Previous studies have demonstrated that Fe(III)PP has low solubility in water (<5%) at pH 2–5.5 but dissolves well and rapidly below pH 2 due to the presence of ferric ions and formation of soluble ferric pyrophosphate complexes.^15−17^ However, it has been observed that even though poorly water-soluble Fe(III)PP causes fewer changes in the organoleptic properties, it does not fully prevent the iron-mediated reactions when added to phenolic-rich foods within the human diet pH range.^18,19^ Additionally, poorly water-soluble Fe(III)PP has the drawback of low iron bioavailability, leading to inadequate iron uptake in the human body.^20,21^ In contrast, Fe(II) sources such as ferrous sulfate and ferrous fumarate have high iron bioavailability.^14^ However, these Fe(II) sources are not ideal for foods that are highly sensitive to color and flavor changes due to their high solubility, which increases iron reactivity with food.^22^ On the other hand, encapsulation of these iron sources is often not preferred due to high costs.^22−25^

Among iron(II) sources, not much research has been done on the iron(II) pyrophosphate (Fe_2_P_2_O_7_, Fe(II)PP) salt. Fe(II)PP has been studied as anode material for lithium-ion batteries due to its relatively higher specific capacity and better cyclic performances.^26−28^ However, their potential applications in food have not been investigated as far as we know. While Fe(III)PP, which has an off-white color compared to the green Fe(II)PP, is preferred for food applications, the advantage of using Fe(II)PP is delivering iron in ferrous form, which has been shown to be absorbed significantly better by the human body compared to ferric.^29^

It has been previously shown that the inclusion of a second divalent metal, such as Ca alongside iron(III) in a pyrophosphate matrix, enhances the iron solubility under gastric conditions (pH 1–3) up to 4-fold, while the iron solubility decreases down to 8-fold in food pH (3–7) compared to Fe(III)PP.^16^ Furthermore, van Leeuwen et al. demonstrated that incorporating an excess of a divalent metal (M) like Mg in Fe(III)PP (Mg/Fe(III) ratio 50:1) hinders its reactivity with phenolic compounds.^30^ Interestingly, the divalent metal pyrophosphate salts, such as calcium pyrophosphate (CaPP), zinc pyrophosphate (ZnPP), and manganese(II) pyrophosphate (MnPP), are poorly water-soluble/insoluble but dissolve well in inorganic mineral acids.^31,32^ In light of these observations, we hypothesize that combining a second divalent metal with Fe(II) in a pyrophosphate matrix would result in low water solubility as well while still exhibiting higher iron solubility in gastric pH (1–3). In addition to reducing the reactivity of iron by embedding it into another less chemically reactive and more preferred white/off-white mineral carrier, these compounds have the advantage of being promising dual-fortificants with adjustable composition of a second essential mineral, such as calcium, zinc, or manganese, along with iron.

Inadequate intake of vitamin C from fresh fruits and vegetables is one of the risk factors for developing iron deficiency.^33^ Ascorbic acid, another name for vitamin C, is a water-soluble vitamin that significantly improves the absorption of most iron compounds and nonheme iron from food.^34^ Therefore, the addition of vitamin C to iron-fortified food products has attracted a great deal of attention. Consequently, there has been a great deal of interest in vitamin C added to dietary products that are fortified with iron. Ascorbic acid is added to oils, fats, and soft drinks to improve iron absorption, in addition to cocoa products, which are recommended as dietary vehicles for iron and vitamin C fortification.^33^ Ascorbic acid is, however, relatively unstable in humid environments and/or high temperatures as well as in the presence of oxygen and metals. In biological systems, vitamin C functions as a scavenger of free radicals and can oxidize to produce dehydroascorbic acid (DHA).^35^ This oxidation process, referred to as the Fenton reaction^36^ and caused by the loss of ascorbic acid in iron-rich diets,^37^ is facilitated by transition metals, particularly cupric (Cu(II)) and ferric (Fe(III)). Moreover, ascorbic acid can induce Fenton-like reactions in foods that result in the development of off-colors and off-flavors.^38^ This pro-oxidant effect occurs when the level of available ascorbic acid is relatively low and insufficient to scavenge the radicals created by Fenton-like processes. Reactive oxygen species (ROS) and transition metals are the main mechanisms responsible for the oxidative loss of ascorbic acid in both dietary and physiological situations. Nevertheless, the precise mechanisms, rate constants, and stoichiometric ratios for the reactions involving transition-metal reactions remain unknown to date.^37^ The majority of research has demonstrated that supplementing diets with iron and vitamin C at a 2:1 mol ratio (6:1 weight ratio) will greatly improve the absorption of iron in foods for both adults and children.^33^ However, a significant drawback of ascorbic acid as a food ingredient in iron-fortified products containing Fe(III) salts is that a significant portion of it may be lost during food preparation and storage.^39^

In this study, we establish the potential application of Fe(II)PP in food fortification. Additionally, we explore the possibility of combining Fe(II) with a second divalent metal in a pyrophosphate matrix with the general formula M_2(1–*x*)_Fe_2*x*_P_2_O_7_ (where 0 < *x* < 1 and M = Ca, Zn, or Mn), which serves as a simultaneous delivery system for two essential minerals. To achieve this, we use a fast, easy, and environmentally friendly coprecipitation method to integrate iron(II) and the second divalent metal homogeneously into the pyrophosphate matrix structure. It is important to note that when fortifying food products, precautions must be taken to avoid any metal toxicity. Therefore, the ratio of the second mineral M to Fe should be limited according to the recommended daily allowance (RDA) for each mineral and should not exceed the tolerable upper intake level (UL), which is defined as the highest level of daily nutrient intake that is likely to pose no risk of adverse health effects to almost all individuals in the general population.^40^ The RDA for each essential mineral, which may depend on the country, is usually regulated much lower than the UL. As intake increases above the UL, the risk of adverse effects increases. The amount of essential minerals used in foods usually does not exceed 30% of the RDA, which assures no adverse effects or toxicity.^40^ After characterization and determination of the final chemical composition, we explore the pH-dependent dissolution behavior of both the pure and mixed pyrophosphate salts. To ensure adequate bioaccessibility of iron from these mixed Fe(II)-containing salts, their iron solubility under gastric-mimicked conditions (incubated at pH 2 and 37 °C for 75 min) is investigated as well. Finally, we test the designed salts in two ways: (i) their Fe-mediated discoloration of a black tea solution, which serves as a representative phenolic-rich model solution, and (ii) their Fe-mediated oxidation of vitamin C, compared to the autoxidation of this vitamin in pure water.

## 2 Materials and Methods

### 2.1 Materials

Tetrasodium pyrophosphate decahydrate (Na_4_P_2_O_7_·10H_2_O, >99 wt %), calcium dichloride (CaCl_2_, >93 wt %), manganese(II) chloride (MnCl_2_, ≥96 wt %), nitric acid (HNO_3_, 65 wt %), 3-(2-pyridyl)-5,6-diphenyl-1,2,4-triazine-*p*,*p*′-disulfonic acid monosodium salt hydrate (ferrozine; ≥97 wt %), hydrochloric acid (HCl, 37 wt %), sodium hydroxide (NaOH, ≥98 wt %), 1,2 phenylenediamine (OPDA), and dehydroascorbic acid (DHA) were obtained from Sigma-Aldrich (St. Louis, MO). Iron(II) sulfate heptahydrate (FeSO_4_·7H_2_O, ≥99 wt %) and anhydrous zinc chloride (ZnCl_2_, ≥98 wt %) were obtained from Alfa Aesar (Haverhill, MA). Ethanol absolute (≥99 wt %) and ascorbic acid (vitamin C, ≥99 wt %) were obtained from VWR International (Radnor, PA). The Milli-Q (MQ) water used was deionized with a Millipore Synergy water purification system (Merck Millipore, Billerica, MA). The tea used for the reactivity assessment was an Original English tea blend from Pickwick (Amsterdam, The Netherlands).

### 2.2 Preparation, Characterization, and Dissolution

Pure divalent metal pyrophosphate salts: iron(II) pyrophosphate (Fe_2_P_2_O_7_, Fe(II)PP), calcium pyrophosphate (Ca_2_P_2_O_7_, CaPP), zinc pyrophosphate (Zn_2_P_2_O_7_, ZnPP), and manganese(II) pyrophosphate (Mn_2_P_2_O_7_, MnPP), were synthesized. Furthermore, three different series of mixed Fe(II)-containing pyrophosphate salts were prepared, each with a different second divalent metal (M) and different M and Fe(II) contents, based on the general formula M_2(1–*x*)_Fe_2*x*_P_2_O_7_ (0 < *x* < 1), for theoretical *x*-values (0.05, 0.10, 0.25, and 0.50, coded as MMix1 to MMix4, where M = Ca, Zn, or Mn). The preparation of the salts was done via a well-established fast, facile, and nonpolluting coprecipitation method, which has been described elsewhere previously^16,41,42^ and is described in detail in the Preparation Methods Section of the Supporting Information. The average yields for the pure salts were 88% for Fe(II)PP, 74% for CaPP, 80% for ZnPP, and 72% for MnPP, and for the mixed salts with M = Ca, Zn, and Mn, the average yields were 33 ± 3, 69 ± 10, and 82 ± 7%, respectively. Standard deviations were calculated based on three independent syntheses of the mixed salts.

The colors of the dried powders of all of the mixed as well as the pure salts were visualized and quantified by taking an image of them illuminated with a uniform light source and using an online color conversion tool to the L*a*b* color space values (L* dark or light, a* red vs green, b* yellow vs blue).^16^ The colors of the pure salts were dark green for Fe(II)PP and white/off-white for all other salts (CaPP, ZnPP, and MnPP). Furthermore, the mixed salts were crème/light yellow when M = Ca, light/dark green when M = Zn, and orange/brown when M = Mn, as shown in Figure S1 of the Supporting Information.

Moreover, the morphology of the salts was investigated by transmission electron microscopy (TEM) on the dried water dispersions of the samples. TEM combined with energy-dispersive X-ray spectroscopy (TEM–EDX), elemental mapping in high-angle annular dark-field scanning TEM (HAADF–STEM), or inductively coupled plasma atomic emission spectroscopy (ICP-AES) were performed to obtain the chemical composition of the salts; for details, see Characterization S1–S3 of the Supporting Information. Based on the general formula of the mixed salts, the elemental composition of the salts was derived and utilized to obtain the experimental *x*-value. In order to determine the compositions, *x* in the structural formula M_2(1–*x*)_Fe_2*x*_P_2_O_7_ was found using the ratios of the atomic percentages (M/Fe = 2(1 – *x*):2*x*, M/P = 2(1 – *x*):2, Fe/P = 2*x*:2). It is important to note that the *x*-values in the general formula represent the minerals (M and Fe(II)) or, in other words, the chemical composition of the salts. The average *x*-value for each mixed salt was reported with a standard deviation based on 3 replicate preparations of the salts and 3 independent measurements. Furthermore, the crystalline structure and chemical bonds of the pyrophosphate salts were investigated by X-ray diffraction (XRD) and Fourier transform infrared (FT-IR) spectroscopy, respectively (Characterization S4 and S5 of the Supporting Information).

Additionally, the pH-dependent dissolution behavior of the pure and mixed divalent metal Fe(II)-containing pyrophosphate salts was explored over pH range 1–11 (steps of two pH units), after incubating for 2 h at 23 °C, according to a previously reported method,^16^ and quantified by a ferrozine-based colorimetric assay^43^ and inductively coupled plasma atomic emission spectroscopy (ICP-AES) (Dissolution S1 of the Supporting Information). The dissolution behavior of all of the pyrophosphate salts was studied under gastric-mimicked conditions (incubation at pH 2 and 37 °C for 75 min) as well, as shown in Dissolution S2 of the Supporting Information. Evaluation of the significance of differences in element concentrations was carried out by statistical analysis (significant at *p* < 0.05).

### 2.3 Reactivity Assessment in a Black Tea Solution

To assess the reactivity of iron in the mixed divalent metal pyrophosphate salts, a black tea solution was used as a representing model system for phenolic compounds (catechins). The procedure was similar to a previously reported work.^16^ Boiling MQ water was mixed with ground tea leaves of Pickwick Original English blend (final concentration 1 g/100 mL). The tea leaves were removed using 1541-125 cellulose filter papers (Whatman, Maidstone, U.K.) after stirring for 3 min. The Fe(II)-containing salts with a normalized concentration of iron (1.05 mg of Fe in 100 mL of tea) were added to the tea solution. For comparison, CaPP, ZnPP, or MnPP with a normalized concentration of 1.05 mg Ca, Zn, or Mn in 100 mL tea was added to the tea solution as well. The discoloration of the filtered tea solutions was recorded on a Lambda-35 spectrophotometer (PerkinElmer, Waltham, MA) at room temperature. The increase in the area under the curve in the visible wavelength range (AUC_380–700_) was compared to the blank black tea solution and used to quantify the iron-phenolic complexation.^44^ All of the measurements were done in independent duplicates, and the average values along with standard deviations were reported. The colors of the tea solutions were converted to the L*a*b* color space (see Section 2.2), and the color difference of the tea solution between after and before being exposed to the salts was quantified using ΔE* as a numerical tool^45^ and is described in detail in the Color Difference Section of the Supporting Information. The concentration of the iron released from the salts in the tea solutions was measured as described in the Dissolution Methods Section of the Supporting Information. A Lambda-35 spectrophotometer (PerkinElmer, Waltham, MA) was used to measure the absorbance of total Fe at 565 nm at room temperature. Statistical analysis was used to determine the significance of variations in iron concentration (significant at *p* < 0.05).

### 2.4 Oxidation of Vitamin C in the Presence of the Salts

To study the oxidation of vitamin C in the presence of the salts, an aqueous solution of vitamin C (ascorbic acid) was added to the dispersions of the salts in MQ water to reach final concentrations of 10 mg/mL and 200 mM (at least 3 times in excess) for the salts and vitamin C, respectively. The dispersions were then incubated under continuous stirring at 1000 rpm and 23 °C for 1, 2, and 48 h. After measuring the final pH of the dispersions, the supernatants were isolated by centrifugation to quantify the total dissolved Fe and Fe(II) (see Dissolution S2 of the Supporting Information) and dehydroascorbic acid (DHA) concentrations by an OPDA-based fluorometric assay.^35^

#### 2.4.1 Quantification of Dehydroascorbic Acid (DHA) Concentration by an OPDA-Based Fluorometric Assay

**Figure 1:**
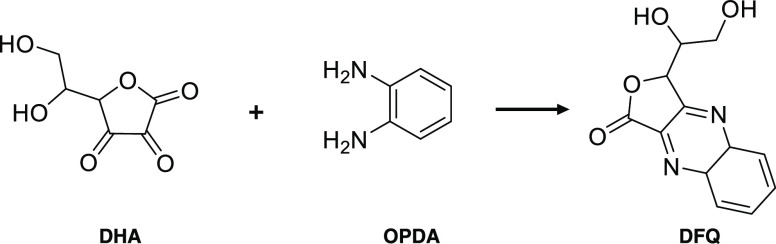
Reaction between DHA and OPDA. Dehydroascorbic acid (DHA) and 1,2 phenylenediamine (OPDA) react with a 1:1 mol ratio and form 3-(1,2-dihydroxyethyl)-fluoro[3,4-*b*]quinoxaline-1-one (DFQ), which can be detected by fluorescence spectroscopy and related to the oxidation extent of ascorbic acid.

An OPDA-based fluorometric assay was utilized to monitor the content of dehydroascorbic acid, or DHA, the oxidation product of vitamin C.^35^ Under acidic conditions, DHA reacts with 1,2 phenylenediamine (OPDA) and forms 3-(1,2-dihydroxyethyl)-fluoro[3,4-*b*]quinoxaline-1-one (DFQ), Figure 1, which is a highly fluorescent compound and can be quantified by fluorescence spectroscopy with excitation and emission wavelengths of approximately 365 and 430 nm, respectively.^35,46,47^ Due to the 1:1 stoichiometric ratio between DHA and OPDA, changes in DFQ concentration can be linked to changes in DHA concentration and, hence, oxidation extent of vitamin C.

Following the separation of the supernatants, a 100 μL sample was mixed with a 0.1 M HCl solution containing 200 mM of OPDA. A 30 min reaction time was selected in order to guarantee the full reaction of DHA with OPDA.^35^ The samples were then transferred to a 384-well black plate, and the fluorescence at 425–435 nm was recorded at room temperature by a CLARIOstar Plus Microplate Reader (BMG LABTECH, Ortenberg, Germany). All measurements were performed in duplicate, and quantification of total DHA was performed based on intensity with a calibration curve of DHA (0.001–1 mM, *R*^2^ > 0.99). The concentration of DHA after exposure of vitamin C to the salts was normalized with respect to the concentration of DHA in the absence of the salts (the autoxidation product of vitamin C in pure water). Evaluation of the significance of differences in DHA concentration was carried out by statistical analysis (significant at *p* < 0.05).

## 3 Results and Discussion

### 3.1 Chemical Composition of the Mixed Divalent Metal Pyrophosphate Salts

The morphology, crystallinity, and chemical bonding of the mixed divalent metal pyrophosphate salts as well as the pure salts (Fe(II)PP, CaPP, ZnPP, and MnPP) were investigated in detail, as shown in Figures S3–S5 of the Supporting Information. Furthermore, the chemical compositions of the mixed divalent metal Fe(II)-containing pyrophosphate salts, designed with *x* = 0.05, 0.10, 0.25, and 0.50 in the general formula M_2(1–*x*)_Fe_2*x*_P_2_O_7_ (coded as MMix1–4, where M = Ca, Zn, or Mn), were characterized by TEM–EDX and ICP-AES (Table 1).

**Table 1:** Measured *x*-Value for the Mixed Fe(II)-Containing Pyrophosphate Salts with General Formula M_2(1–*x*)_Fe_2*x*_P_2_O_7_, Obtained from EDX (for M = Ca and Zn) and ICP-AES (for M = Mn) Quantification^a^

second divalent metal (M)	designed *x*	0.05	0.1	0.25	0.5
M = Ca	salt code	CaMix1	CaMix2	CaMix3	CaMix4
	measured *x*	0.060 ± 0.002	0.130 ± 0.003	0.240 ± 0.030	0.470 ± 0.060
M = Zn	salt code	ZnMix1	ZnMix2	ZnMix3	ZnMix4
	measured *x*	0.047 ± 0.006	0.074 ± 0.004	0.202 ± 0.001	0.470 ± 0.030
M = Mn	salt code	MnMix1	MnMix2	MnMix3	MnMix4
	measured *x*	0.053 ± 0.002	0.086 ± 0.002	0.220 ± 0.010	0.520 ± 0.040 (R)
					0.350 ± 0.020 (I)

a The measured *x*-values for all of the mixed salts were close to the designed values, except in the case of MnMix4. This salt shows different measured *x*-values for the coexisting morphologies, (R) rod shape and (I) irregular shape.

The measured *x*-values obtained by EDX for the salts CaMix1–4 were rather close to the designed *x*-values, measured *x* vs designed *x* for M = Ca in Table 1. The EDX measurements for M = Zn showed that the measured *x*-values in all of the mixed salts were, on average, somewhat lower than the expected (planned) *x*-values; these values were, in order, 0.047, 0.074, 0.202, and 0.47 for the salts ZnMix1–4 in Table 1. Furthermore, the case in which M = Mn indicates that the average *x*-values for salts MnMix1–3 were found (by ICP-AES) to be approximately 0.053, 0.086, and 0.220, respectively. The salt MnMix4 comprised of local segregation and coexistence of two morphological phases: a rod-shaped phase with a much higher Fe content (higher *x*-value) and an irregularly shaped phase with a lower Fe content (lower *x*-value), as shown in Figures S3C and S4C of the Supporting Information. The letters R and I correspond to rod shape and irregular shape, respectively, for MnMix4 in Table 1. This salt seemed to include a combination of a phase rich in iron with *x* = 0.520 and a phase rich in manganese with *x* = 0.350. Phase separation in the ensuing solid solutions may account for producing the Fe-rich and Mn-rich phases of particles, which result in different observed *x*-values (from EDX quantification).^48,49^ Hereafter, for the sake of convenience, the average of these two *x*-values is supplied for additional research (for MnMix4, the measured *x* = 0.44).

### 3.2 pH-Dependent Dissolution Behavior of the Pure and Mixed Divalent Metal Pyrophosphate Salts

**Figure 2:**
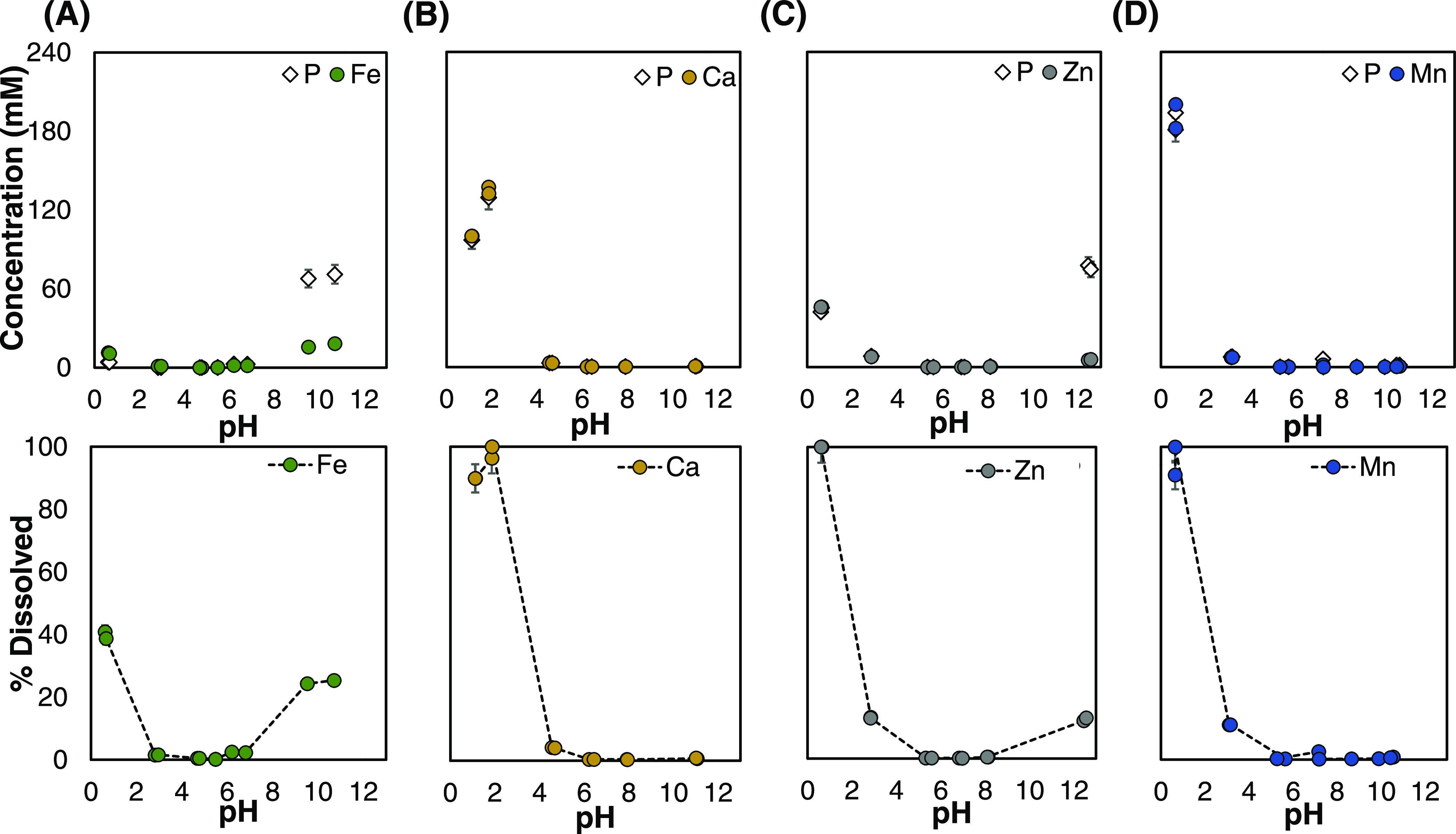
pH-dependent dissolution behavior of the pure divalent metal pyrophosphate salts obtained by ICP-AES. Concentrations of the elements (top) and percentage of the dissolved metal (bottom) for (A) Fe(II)PP, (B) CaPP, (C) ZnPP, and (D) MnPP.

One of the primary difficulties in designing iron-containing compounds for food fortification is ensuring that the substance dissolves slowly in the food pH range (3–7) and quickly and readily in the gastrointestinal pH range (1–3 and 6–8). Initially, the ICP-AES method was utilized to assess the pH-dependent dissolution behavior of the pure divalent metal pyrophosphate salts, including Fe(II)PP, CaPP, ZnPP, and MnPP (Figure 2).

Here, we present, for the first time, the pH-dependent dissolution behavior of Fe(II)PP. Quantification of the element (Fe and P) concentrations from the Fe(II)PP showed that interestingly this salt has limited solubility in pH 3–7 and high and/or fast dissolution at pH < 3 and > 7 (Figure 2A (top)). Results showed that Fe(II)PP remained (practically) insoluble (<0.1 g/L^50^) in the pH range 3–5 with a maximum of 1.5% dissolved iron, compared to the total initial iron present in the salt (Figure 2A (bottom)). Furthermore, the soluble iron concentrations from this salt in pH 5–7 were measured to be lower than 1.7 mM, being a maximum of 2.5% of the total initial iron. It has previously been reported that the soluble iron concentration from Fe(III)PP in the same pH range can reach up to approximately 3 mM.^16^ Therefore, our findings suggest that Fe(II)PP shows less solubility and is expected to have potentially less iron-mediated reactivity within pH 5–7, compared to the previously studied Fe(III)PP.^9,15,16,30,41^

The soluble iron concentration from Fe(II)PP increased to around 11 mM at pH 1 (approximately 40% of the initial iron). At pH range below 3, the dissolution of iron from Fe(II)PP may occur because of soluble ferrous pyrophosphate complexes such as Fe(H_3_P_2_O_7_)_2_ and Fe(H_2_P_2_O_7_)_2_^2–^,^[Bibr ref]^^[Bibr ref]^ ferric pyrophosphate complexes (due to iron redox reaction) such as FeH_3_P_2_O_7_^2+^ and FeH_2_P_2_O_7_^+^,^52,53^ free iron ions at low pH, and/or possible Fe(II)/Fe(III) chloride complexes with pyrophosphate species (pyrophosphoric acid has p*K*_a1_ < 1 and p*K*_a2_ ≈ 1.5^5,54^).

The concentration of soluble iron increased dramatically above pH 7, reaching 18 mM at pH 10. Soluble iron concentration in pH 7–9 indicated that the dissolved iron from this salt around pH 8 was approximately 7.8 mM. Moreover, it was proposed that the development and sedimentation of iron oxides and hydroxides, which were seen as dark orange/brown sediments in the titrated sample, was the cause of the lower detected Fe content, compared to P, at pH 10–11. A comparison of the dissolution behavior of Fe(II)PP and Fe(III)PP from our previous study^16^ at the gastrointestinal pH range showed that the soluble iron from Fe(II)PP was higher than Fe(III)PP up to 11 and 2.6 times at pH 1 and pH 8, respectively. These results imply that Fe(II)PP, as opposed to Fe(III)PP, is a viable option for iron fortification of foods with a wider pH range and higher and/or faster dissolution at gastrointestinal-relevant pH.^15,55−58^

As demonstrated by quantification of the metals (Ca, Zn, and Mn) and phosphorus (P) concentrations from the pure pyrophosphate salts, all of the salts had restricted solubilities at pH 4–7 and rapid dissolution in low pH (1–3), as shown in Figure 2B–D. Based on the determination of soluble calcium concentration from CaPP, this salt has limited solubility at pH ≥ 5. At pH 5, its maximum Ca concentration is 3.2 mM, meaning that 4.2% of the total Ca contained in the salt dissolves. The soluble Ca was shown to increase up to about 130 mM at pH 2, which was above 96% dissolution, at pH less than 5. With a maximum concentration of 0.3 mM for Zn at pH 8 or about 0.5% of the total Zn contained in the salt, ZnPP demonstrated poor solubility in pH 5–8. ZnPP is soluble at pH < 5, while the dissolved Zn from this salt reaches >99% at pH 1. Additionally, at pH > 8, the solubility of ZnPP increased to 6 mM at pH 12 or around 13% of the salt’s total zinc content. The color of the remaining silt (dull white) indicated the presence of insoluble zinc oxide and hydroxide, which was again proposed as the cause of the lower Zn concentration measured in relation to P. According to pH-dependent dissolution behavior of MnPP, the dissolved manganese content at pH > 5 was below 2 mM or about 2.5% of the total Mn in the salt. Additionally, when pH < 5, the soluble Mn from MnPP increased to >99% at pH 2.

It has previously been shown that the presence of Ca and Fe(III) in a single pyrophosphate matrix can limit the amount of soluble iron present in mixed salts at pH 3–7. Based on the results obtained from the dissolution of the pure salts, it is hypothesized that adding the second metal (M) to the pyrophosphate matrix along with Fe(II) would also lead to limited dissolution of the mixed compound, at least in pH 5–7, while demonstrating relatively higher soluble iron (and M) at pH < 3.

**Figure 3:**
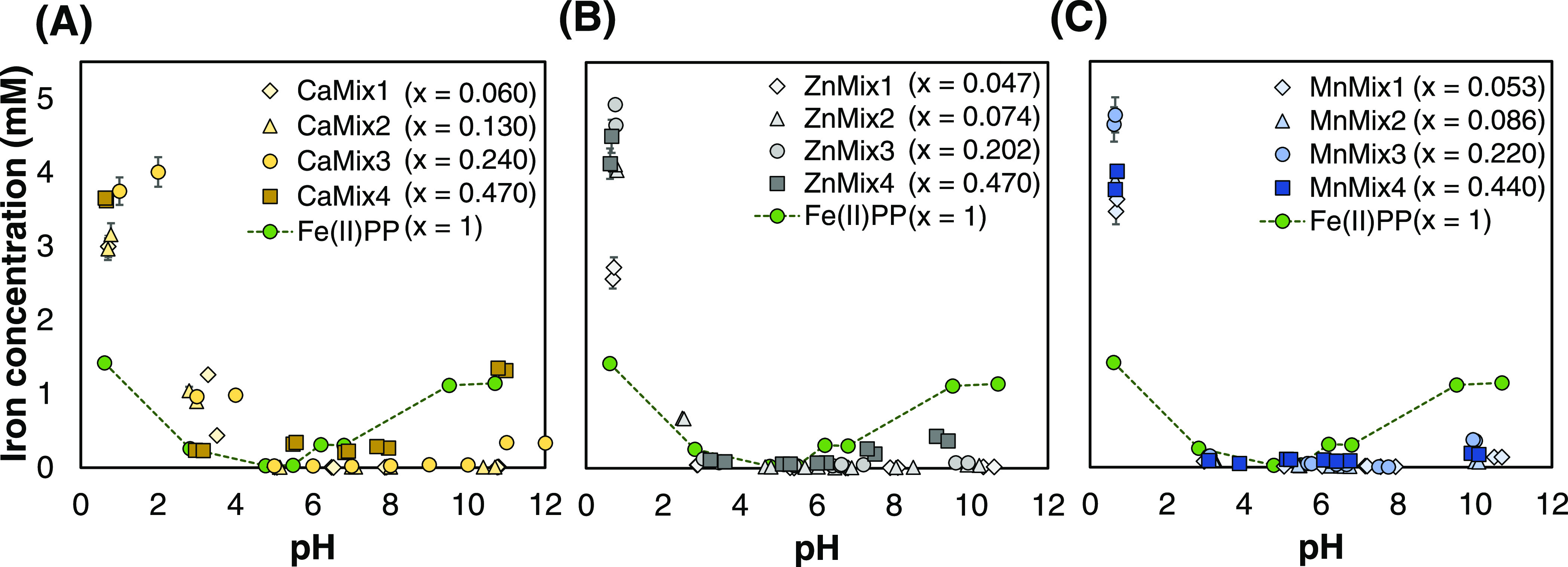
Dissolution behavior of iron from the mixed divalent metal Fe(II)-containing pyrophosphate salts in comparison to Fe(II)PP. Concentration of soluble iron from the mixed M_2(1–*x*)_Fe_2*x*_P_2_O_7_ salts as a function of pH when (A) M = Ca, (B) M = Zn, and (C) M = Mn, after 2 h of incubation at 23 °C. The dissolution profile of iron from Fe(II)PP is shown for comparison (dashed lines). Results clearly show that all of the mixed salts have very limited dissolution of iron in the moderate pH (4–7) while showing enhanced dissolved iron concentration in the gastric pH up to 5 mM.

To assess the plausibility of our hypothesis, the dissolution behavior of iron from the mixed salts was evaluated over pH 1–11 and compared to that of Fe(II)PP (Figure 3). Figure S6 of the Supporting Information confirms that the quantification of total iron in the ferrozine test was not interfered with by the presence of Ca, Zn, or Mn ions. Quantification of the dissolved iron from the mixed salts revealed that, overall, all salts exhibited improved dissolved iron in the gastric and intestinal pH ranges (up to 5 and 1.3 mM, respectively) but very limited iron dissolution (<0.5 mM) in the moderate pH (4–7) (Figure 3A–C). Consequently, it was confirmed that adding Ca, Zn, or Mn, the divalent metals investigated in this work, to the pyrophosphate salt matrix together with Fe(II) produced a distinct dissolution behavior appropriate for iron fortification of foods.

In the case of Ca as the second divalent metal, the dissolved iron concentration from all mixed salts decreased above pH 4.5, compared to that of Fe(II)PP (Figure 3A). CaMix4, with *x* = 0.470, at pH 5.5 and >10 was an exception to this. Comparing the salts CaMix1–3 to Fe(II)PP at pH 4.5–7, the soluble iron concentration of the former was 90-fold lower, with CaMix1 (*x* = 0.060) showing the lowest concentration at pH 6.5. Furthermore, of these salts, the one with the maximum iron dissolution in this pH range was CaMix4 (*x* = 0.470). Additionally, at pH ≤ 4, the mixed salts with M = Ca had a larger dissolved iron concentration than Fe(II)PP; in the case of CaMix3 (*x* = 0.240) at pH 2, this difference was around 16 times. In light of this, it is anticipated that, in comparison to Fe(II)PP, the salts CaMix1–3 display greater bioaccessibility at pH 1–3 and less iron-mediated reactivity at food pH. According to these results, mixed salts with M = Ca and *x* ≤ 0.240 are good candidates for dual-fortificants (calcium and iron), especially in food vehicles with pH 5–7.

Lower dissolved iron was seen for all mixed salts over pH 1–11 in the dissolving behavior of iron from the mixed salts with M = Zn, as compared to Fe(II)PP (Figure 3B). The only exception to this was ZnMix4 (*x* = 0.470) with roughly similar iron dissolution at pH 5. Nevertheless, this salt is practically insoluble. The maximum soluble iron content of this salt was around 0.05 mM (3.2 mg/L). Zn accompanying Fe(II) in the pyrophosphate matrix reduced the soluble iron content of the mixed salts in the pH range relevant to food (3–7). ZnMix1 (*x* = 0.047) had the lowest dissolved iron, measuring 12 and 120 times lower at pH 5.5 and 6.5, respectively, than Fe(II)PP. Additionally, in the case of ZnMix3 (*x* = 0.202) at pH 1 (5 mM), the maximum iron solubility from the mixed salts with M = Zn was 3.5 times higher than Fe(II)PP below pH 3. It is noteworthy that at gastric pH, this salt had the highest concentration of dissolved iron out of all of the mixed divalent Fe(II)-containing salts. Consequently, the mixed salts with *x* ≤ 0.202 and M = Zn seem to be promising candidates for the simultaneous fortification of foods with iron and zinc.

In the case of M = Mn in the mixed salts, the dissolution behavior of iron from the salts was very similar to that of the salts with M = Zn. Overall, these salts had less soluble iron above pH 3 than Fe(II)PP, with the exception of MnMix4, which has dissolved iron that is about 5 times higher at pH 5 (Figure 3C). This salt, like ZnMix4, was practically insoluble (0.1 mM). At pH 3–7, MnMix1 (*x* = 0.053) showed approximately 3–30 times lower dissolved iron than Fe(II)PP at pH 5–6.5. Additionally, compared to Fe(II)PP, the mixed salts with M = Mn demonstrated improved iron solubility below pH 3. The highest amount of soluble iron in this pH range (4.8 mM) was found in MnMix3 (*x* = 0.220), which was around 3.35 times more than Fe(II)PP. According to these findings, mixed salts with M = Mn and *x* < 0.220 make good choices for dual-fortifying of food products with manganese and iron.

### 3.3 Dissolution Behavior of the Pure and Mixed Pyrophosphate Salts under Gastric-Mimicked Conditions

**Figure 4:**
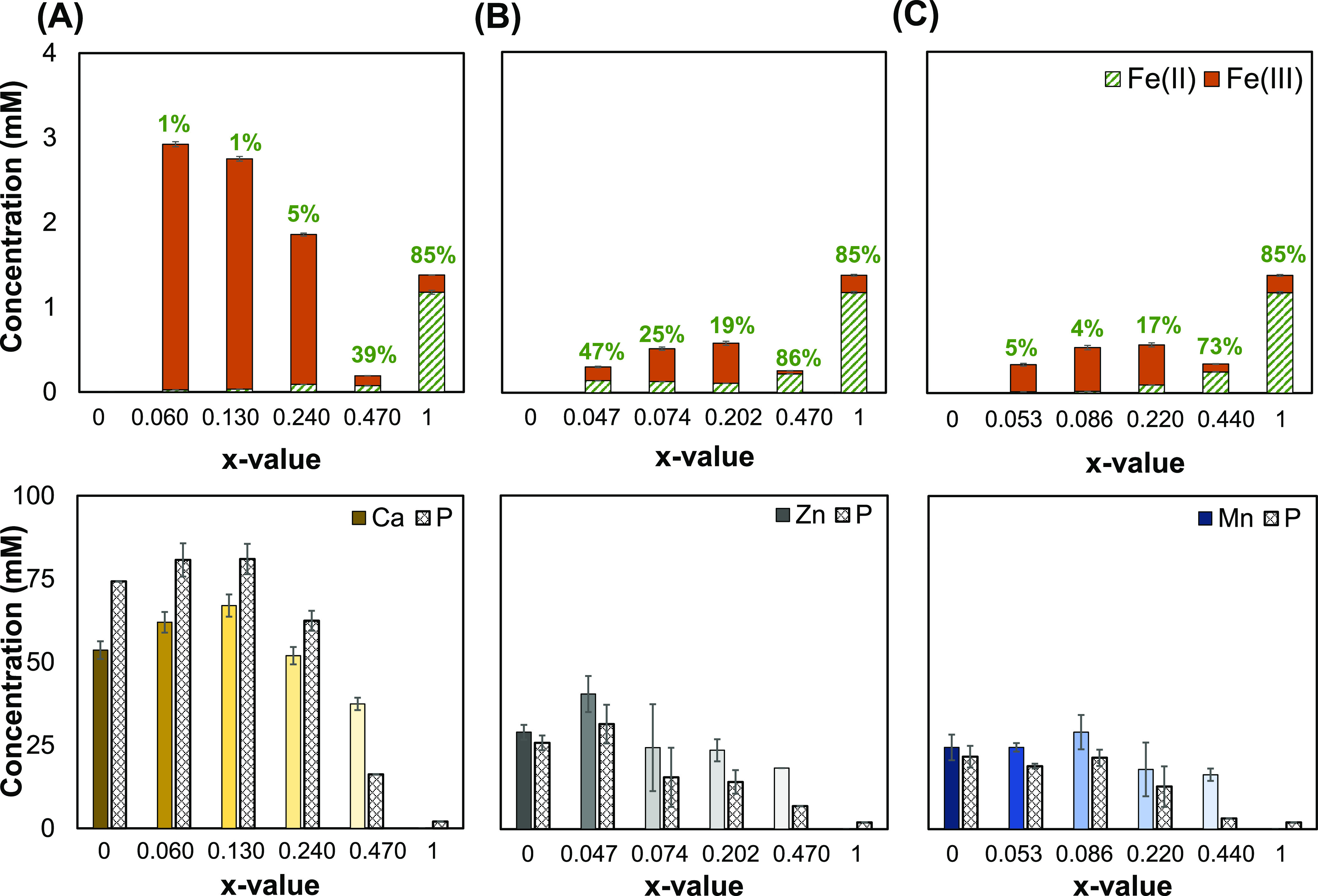
Dissolution behavior of iron from the salts under gastric-mimicked conditions. Quantification of the dissolved iron(II) (green, pattern) and iron(III) (orange, solid) concentrations (top, obtained from UV–vis spectroscopy), as well as the second metal (M = Ca, Zn, or Mn, solid) and phosphorus concentrations (P, pattern) (bottom, obtained form ICP-AES) from the mixed M_2(1–*x*)_Fe_2*x*_P_2_O_7_ salts where (A) M= Ca, (B) M= Zn, and (C) M= Mn under gastric-mimicked conditions. The percentage of Fe(II) with respect to the total iron in solution is written on each column (top).

Even though poorly water-soluble or water-insoluble iron sources can maintain the organoleptic properties of the food because of their low iron reactivity, they are disadvantageous due to low iron bioavailability.^21,59^ It is worth noting here that iron bioaccessibility is necessary but not the only requirement for its bioavailability.^57^ Therefore, it is crucial to make sure that the solubility of the iron supply is relatively high and rapid under gastric and/or intestinal conditions. It has previously been reported that higher concentrations of total soluble iron at pH 1–3 can guarantee sufficient bioaccessibility of iron in the stomach^56,60^ and solubility of Fe-containing salts at pH 1 is a good predictor of in vivo iron uptake by rats.^58^ Therefore, we explored soluble Fe(II) and total Fe concentrations from these salts in solution under gastric-like conditions (incubating at pH 2 and 37 °C for 75 min^61,62^) by the ferrozine assay.^43^ Additionally, Figure S7 of the Supporting Information shows that the accuracy of the total iron estimation utilizing the ferrozine assay was confirmed through comparison with ICP-AES. It was found that the outcomes of both methods showed good agreement. Besides iron, concentrations of Ca, Zn, or Mn and phosphorus (P) were quantified (Figure 4). When the mixed divalent metal Fe(II)-containing pyrophosphate salts were incubated under gastric-relevant conditions, the total dissolved iron content in all of the mixed salts was lower than that of Fe(II)PP, with the exception of the salts containing M = Ca. This may be because the final pH of the dispersions was higher than the aimed pH (≈2.1–3.3). The total dissolved iron from the salts with M = Zn and Mn was lower, while the total dissolved iron from the salts with M = Ca was higher than Fe(II)PP in this pH range, in accordance with the findings of the pH-dependent dissolving behavior (Section 3.2).

The mixed salts with M = Ca showed an increase in total soluble iron for all x-values, except for CaMix4 (*x* = 0.470), in comparison to Fe(II)PP (*x* = 1) (Figure 4A (top)). The maximum soluble iron was measured for CaMix1 (*x* = 0.060), being higher than 60% of the total iron present in the salt (approximately 2.1 times higher than Fe(II)PP). This enhanced iron dissolution can be related to the pH-dependent dissolution behavior of CaPP. We have shown in our previous work that a high solubility of CaPP (>99%) at pH ≤ 3^31^ enhances the soluble iron concentration to 4-fold when mixing Ca and Fe(III) in one pyrophosphate matrix.^16^ Furthermore, when Ca and Fe(II) were combined in a single pyrophosphate matrix, Fe(II) in all mixed salts at pH 2 underwent oxidation and was converted to Fe(III). For CaMix4, a maximum of 39% of total dissolved iron was still present as Fe(II). This may be the result of the higher soluble iron content from these mixed salts, which may be more difficult to oxidize. Furthermore, as shown in Figure 4A (bottom), an examination of the ICP-AES data revealed that over 65% of the initial calcium in the salts dissolved under gastric-mimicked circumstances, with the maximum value of ≈67 mM for CaMix2 (*x* = 0.130) representing over 99% of the total initial Ca.

When M = Zn, ZnMix4 (*x* = 0.470) had a total soluble iron content that was 5.3 times lower than that of Fe(II)PP (Figure 4B (top)). For ZnMix3 (*x* = 0.202), the maximal iron dissolution was found to be 0.58 mM. It is interesting to note that for M = Zn, Figure 4B (top), neither the percentage of Fe(II) nor the total soluble iron from the mixed salts was a monotonic function of their *x*-values. Of the metals examined in this work, Zn inclusion alongside Fe(II) in a single pyrophosphate matrix resulted in the least amount of Fe(II) oxidation. ZnMix4 had the highest proportion of Fe(II) measured at 86%, which was comparable to Fe(II)PP. This can be explained by the antioxidant activity of zinc,^63,64^ which prevents the oxidation of Fe(II) up to a certain extent. Furthermore, quantification of the zinc concentration showed that for all salts, at least 40% of the total zinc present in the salt dissolved (Figure 4B (bottom)). The soluble Zn under gastric-mimicked conditions was the highest for ZnMix1 (40 mM, ≈ 64% of the total initial zinc).

Comparing the mixed salts with M = Mn to Fe(II)PP (Figure 4C (top)) shows that overall the concentration of iron in solution dropped (to 4.2-fold). MnMix1 (*x* = 0.053, 0.33 mM) and MnMix3 (*x* = 0.220, 0.56 mM) had the highest and lowest levels of dissolved iron, respectively. Moreover, Mn added along with Fe(II) showed the least amount of iron oxidation for MnMix4 of all of the mixed salts, measuring 73% Fe(II) of the total dissolved iron. Furthermore, Figure 4C (bottom) shows that at least 31% of the manganese present in the salt dissolved under gastric-mimicked circumstances according to measurement of the soluble manganese using ICP-AES. Finally, the highest soluble Mn concentration was measured to be around 29 mM for MnMix2 (*x* = 0.086) and equivalent to 45% of the initial Mn present in this salt.

In summary, our preliminary studies on the bioaccessibility of the minerals in the M_2(1–*x*)_Fe_2*x*_P_2_O_7_ salts indicate that, when M = Ca, the mixed salt with *x* = 0.060 has the highest soluble iron concentration in gastric-mimicked conditions, increasing by a 2.1 factor when compared to Fe(II)PP. Furthermore, mixed salts with *x* = 0.202 and 0.220, where M = Zn and Mn, respectively, showed maximum and comparable dissolved iron concentration (≈0.6 mM) under these conditions.

### 3.4 Assessment of the Reactivity of the Pure and Mixed Salts by Discoloration of a Black Tea Solution

Although the primary goal of this work is not to fortify tea, we did examine the reactivity of iron in the salts with phenolic compounds (catechins) using a black tea solution as a representative model solution. Due to the fact that the significant amount of phenolic chemicals in black tea can provoke discoloration through iron-mediated complexation and oxidation when present in solutions containing iron.^10,65,66^

**Figure 5:**
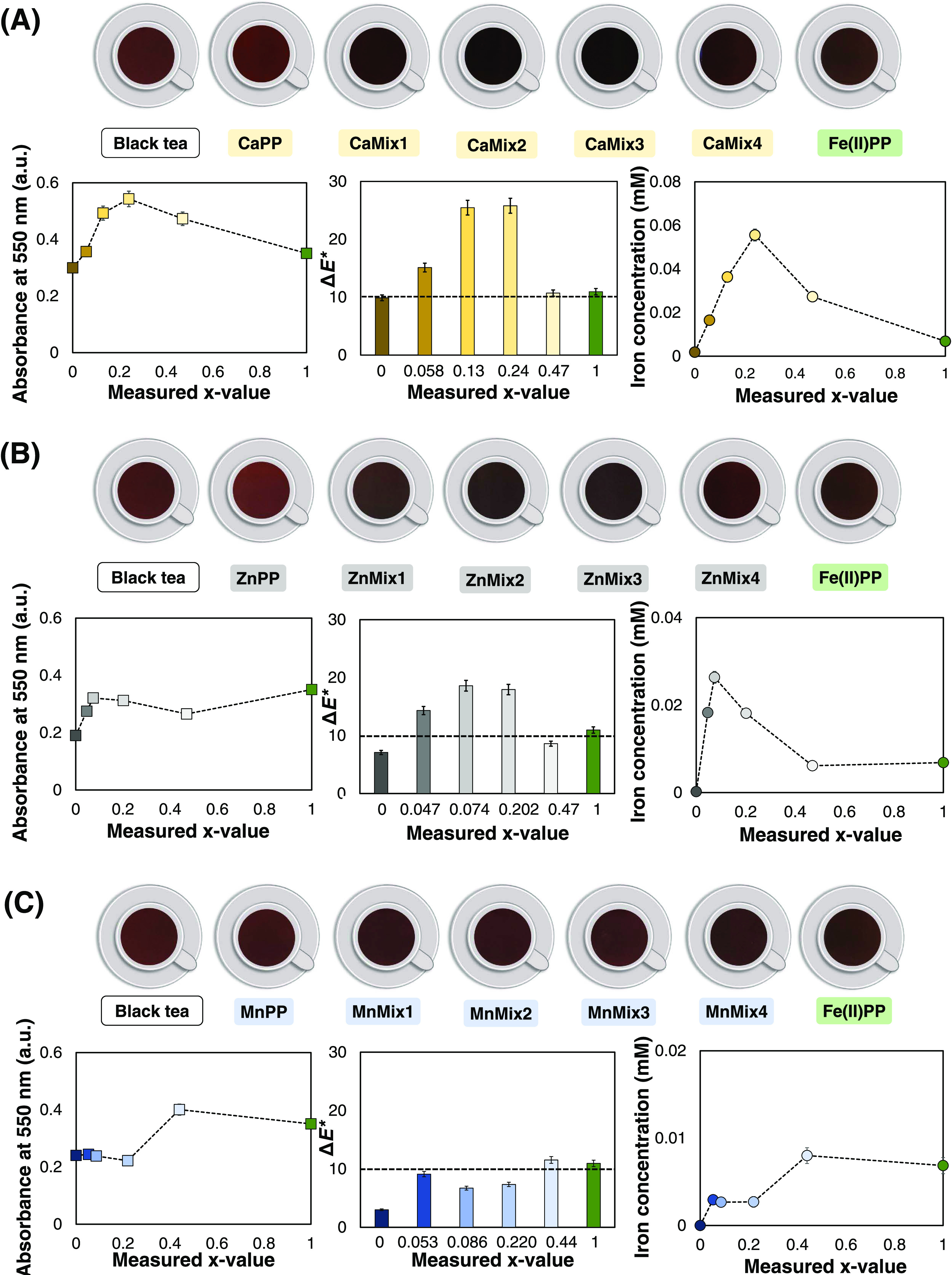
Results of the reactivity assessment by exposing a black tea solution to the mixed M_2(1–*x*)_Fe_2*x*_P_2_O_7_ salts where (A) M = Ca, (B) M = Zn, and (C) M = Mn, compared to Fe(II)PP. Among the mixed divalent metal Fe(II)-containing pyrophosphate salts, (A) CaMix4 (*x* = 0.47) with comparable color difference (ΔE* = 10.7), (B) ZnMix4 (*x* = 0.47) with less color difference (ΔE* = 8.6) and similar concentration of the dissolved iron, and (C) MnMix2 (*x* = 0.086) with less color change (ΔE* = 6.7) and lower iron dissolution (2.3-fold) exhibit less iron-mediated reactivity and discoloration in the black tea solution.

The black tea model solution was exposed to the mixed Fe(II)-containing pyrophosphate salts as well as the pure Fe(II)PP, CaPP, ZnPP, and MnPP salts (final pH of the solutions: 5.2 ± 0.3). Since it was previously proved that these mixed pyrophosphate salts have limited water solubility in moderate pH, it was expected that their mixed salts would exhibit limited iron-mediated solubility and reactivity, resulting in less discoloration. The results of discoloration of the tea solution, caused by iron ions released from the salts, are depicted in Figure 5 (column charts in the middle). The color of the tea solutions was evaluated using the L*a*b* color space to calculate the ΔE* as a measure for color difference of the tea solution before and after being exposed to the salts.^9^ It is assumed that when ΔE* ≈ 3–5, the color difference is observable,^67^ and up to ≈10, the color change in the presence of the iron-fortified salt is acceptable.^68^

The visual color comparison between tea solutions exposed to the mixed salts with M = Ca showed that the color difference, ΔE*, was the highest for CaMix3 (*x* = 0.24) and approximately 2.4 times higher than Fe(II)PP (*x* = 1, ΔE* ≈ 10.9), Figure 5A (column charts in the middle). This was in line with the fact that, among all salts, CaMix3 salt had the highest absorbance at 550 nm^44^ (Figure 5A). Surprisingly, the salt with the highest Fe(II) content, CaMix4 (*x* = 0.47), exhibited comparable discoloration (ΔE* = 10.7) to Fe(II)PP. The illustration of dissolved iron vs the measured *x*-values of these salts showed higher dissolved iron in tea solution up to 8-fold for CaMix3 (*x* = 0.24), compared to Fe(II)PP. This nonmonotonic behavior of discoloration of black tea solution vs their iron content was previously observed for the mixed Ca and Fe(III) pyrophosphate salts as well.^16^ Since the complexation of calcium with phenolics is not known to cause color change,^18^ it is hypothesized that this discoloration is caused by the interaction and oxidation of polyphenols in the presence of calcium at elevated temperatures.^69^

Exposing the black tea solution to the mixed salts where M = Zn (ZnMix1–4) resulted in enhanced discoloration to a maximum 1.7-fold for ZnMix2 (*x* = 0.07), in comparison to Fe(II)PP (Figure 5B). Similar to the case of M = Ca, the ΔE* value was the lowest in the case of ZnMix4 (*x* = 0.47), (ΔE* ∼ 8.6). In line with the results of the color difference, the dissolved iron in the tea solution was maximum for ZnMix2 (*x* = 0.07), approximately 3.8 times higher than that of Fe(II)PP. This quantity was measured to be minimum for ZnMix4 (*x* = 0.47), among all salts, and comparable to Fe(II)PP (0.0061 vs 0.0068 mM) (Figure 5B). These results suggest that the salt ZnMix4 is a potential dual-fortificant for Fe(II) and Zn, which likely shows less discoloration in the presence of the catechins. Interestingly, exposing the black tea to ZnPP (*x* = 0) caused a slight lightening in the black tea solution (L* = 24 for ZnPP vs 20 for pure black tea). This could be explained by the possible complexation of zinc with catechins (characteristic absorbance <350 nm),^70,71^ resulting in light, yellow-colored complexes.^72^ Moreover, it is expected that the oxidation of the tea phenolics is limited after exposure to ZnPP due to antioxidant activity of zinc.^63,64^

Figure 5C displays the outcomes of the discoloration of the black tea solution following exposure to salts containing M = Mn (MnMix1–4). Overall, results showed that the inclusion of Mn in the pyrophosphate matrix successfully caused less discoloration of the black tea solution, indicating the limited iron-mediated reactivity, compared to Fe(II)PP. All of these mixed salts had lower absorbance at 550 nm, lower ΔE* values, and lower dissolved iron concentration in solution when compared to Fe(II)PP. The salt MnMix4 was the only exception to this, Figure 5C (column charts in the middle). It was expected (according to the results of Section 3.1) that MnMix4 would not be a suitable candidate for the purpose of this work due to its heterogeneous morphology and the anisotropy in iron distribution in its matrix. It is worth reminding here that the coexisting morphologies in this salt, one of which possesses higher iron content, are expected to have more available iron for exposure to the phenolics in tea solution, in comparison to the homogeneous aggregates. Moreover, among all salts with M = Mn, the salt MnMix2 (*x* = 0.087) with ΔE* = 6.7 exhibited the least discoloration. In addition, in comparison to Fe(II)PP, the dissolved iron concentration from these mixed salts in the tea solution dropped to 2.3 times for MnMix2 (*x* = 0.087). In light of their iron-mediated reactivity with the catechins of black tea solution, the mixed salts with Mn as the second divalent metal and ≤0.220 seem to be the best options.

To summarize, according to our research, mixed pyrophosphate salts that contain Fe(II) are potential choices for dual-fortification that provides modifiable mineral compositions. The current study’s results show that the best options for simultaneous fortification with F(II) and Ca, Zn, or Mn, respectively, are the mixed salts CaMix4 (*x* = 0.470), ZnMix4 (*x* = 0.470), and MnMix2 (*x* = 0.086). The main benefits of these salts are that they minimize the dissolved iron concentration and reduce iron-mediated discoloration of foods containing catechins. When compared to Fe(II)PP, these salts display iron-mediated discoloration of a black tea solution that is comparable to or less pronounced.

### 3.5 Oxidation of Vitamin C in the Presence of the Pure and Mixed Divalent Fe(II)-Containing Pyrophosphate Salts

We investigated the potential use of Fe(II)PP and the mixed divalent metal Fe(II)-containing pyrophosphate salts M_2(1–*x*)_Fe_2*x*_P_2_O_7_ (where 0 < *x* < 1 and M = Ca, Zn, or Mn) for food fortification in combination with vitamin C, with the goal of achieving higher amounts of soluble iron and intact vitamin C that are accessible for absorption in the human body. This is to address the issue of vitamin C loss from the iron-fortified food products during storage and preparation. In order to achieve this, we contrasted the autoxidation of vitamin C in pure water with the iron-mediated oxidation of vitamin C in water dispersions of these salts.

**Figure 6:**
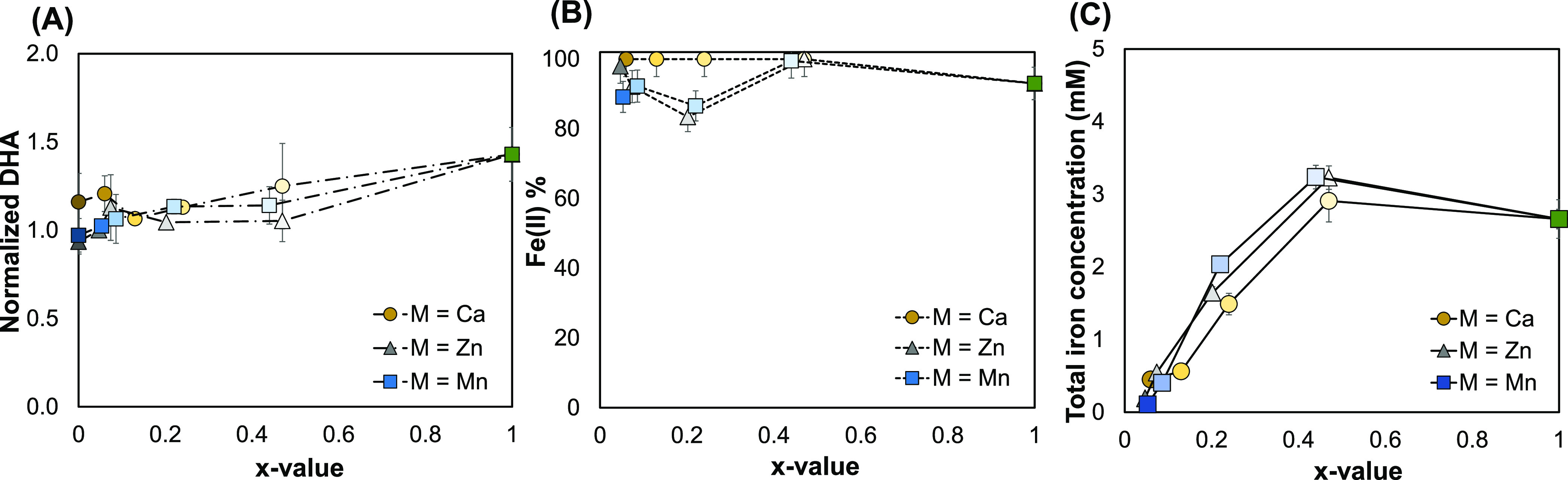
Oxidation of vitamin C in the presence of Fe(II)PP and the mixed divalent metal Fe(II)-containing salts. (A) Normalized DHA (the ratio of the produced DHA concentration in the presence of the salts to the absence of the salts), (B) percentage of the dissolved iron(II), and (C) the total dissolved iron concentration from Fe(II)PP and the mixed salts M_2(1–*x*)_Fe_2*x*_P_2_O_7_ (where 0 < *x* < 1 and M = Ca, Zn, or Mn) after 2 h of incubation at 23 °C.

The results obtained from exposure of the salts M_2(1–*x*)_Fe_2*x*_P_2_O_7_ (0 ≤ *x* ≤ 1, where M = Ca, Zn, or Mn) to vitamin C are illustrated in Figure 6. It should be mentioned that the final pH of the samples dropped to 2.4–3.6, which is in the pH range of foods that are rich in vitamin C.^73^

The generation of 3.8 mM DHA, which was only 1.4 times higher than DHA from the autoxidation of ascorbic acid in water, *x* = 1 in Figure 6A, was a consequence of exposing Fe(II)PP to ascorbic acid in water for 2 h at 23 °C. Additionally, taking into account the 1:1 stoichiometry of ascorbic acid to DHA in the oxidation reaction, this amounted to just 2% of the initial concentration of vitamin C in the dispersion. This indicates that a significant portion of vitamin C in the presence of Fe(II)PP remained intact (about 98%) in solution. Over 48 h, this amount remained stable around 96% (Figure S8 of the Supporting Information). Furthermore, the Fe(II)% and the total soluble iron measured from this salt were ≈93% and 2.6 mM, respectively (Figure 6B,C). This is because the oxidation of iron in solution is prevented by the presence of vitamin C. Moreover, the dissolving tendency at this pH explains the higher soluble iron concentration in the presence of vitamin C as compared to its absence. According to our research, adding Fe(II)PP as an iron-fortificant in a food vehicle (pH ≈ 3) with vitamin C is anticipated to increase the amount of iron(II) available while retaining a significant amount of vitamin C.

Furthermore, the oxidation of vitamin C in the presence of CaPP was slightly higher (1.2-fold), compared with its autoxidation. However, in the case of ZnPP and MnPP, the values for the normalized DHA were approximately 1 (Figure 6A). Therefore, it can be concluded that the presence of these salts had nearly zero effect on the oxidation of vitamin C over 2 h (and 48 h; Figure S8 of the Supporting Information).

In the case of the mixed salts, the average concentration of DHA decreased for all of the salts except for CaMix4, compared to Fe(II)PP, up to roughly 1.43-fold for ZnMix1, which is equivalent to ≈99% intact vitamin C (Figure 6A). Additionally, the presence of the salts with M= Zn resulted in minimum oxidation of vitamin C among all salts (M = Ca, Zn, and Mn). In the cases of ascorbic acid, Zn(II) can form complex with ascorbate ion,^74^ which can stabilize vitamin C similar to how Zn antagonizes the catalytic properties of the redox-active transition metals in the human body.^75,76^

Fe(II)% quantification revealed that almost all of the dissolved iron from all of the salts was in the Fe(II) form when M = Ca. The Fe(II) percentage, however, was lower for the salts when M = Zn and Mn; it was as low as 83.4% for ZnMix3 and 86.6% for MnMix3. However, the ferrozine-based colorimetric assay’s uncertainties may also be the cause for these low Fe(II)% readings.

The dissolved iron from the mixed salts in the vitamin C-containing solution was significantly less than that from Fe(II)PP under the same conditions, even though the total iron concentrations from the mixed salts increased after exposure to vitamin C (because of the lower final pH). This helps to avoid Fe-mediated interactions with dietary constituents that alter the organoleptic properties (Figure 6C). Lastly, the dissolution behavior at similar final pH values when incubated with ascorbic acid (Figure 6C) explains the similarities in the total soluble iron from the mixed Fe(II)-containing salts at nearly identical *x*-values.

Furthermore, to gain more insight into the oxidation rate of vitamin C when exposed to these salts during storage, we explored the retention of vitamin C over time (Figure S8 of the Supporting Information). The normalized DHA concentration when exposing vitamin C to Fe(II)PP showed no significant difference (*p* < 0.05) and fluctuated in the range of 1.1–1.5 over time. Moreover, results indicated that in the case of the mixed salts, the DHA concentration was always measured to be less than 2 times higher for all of the mixed salts, compared to their absence. Following a 48 h incubation period, the mixed salts with *x* = 0.130, 0.470, and 0.440 for M = Ca, Zn, and Mn, respectively, were found to have the highest DHA value of all of the salts. This indicates that about 96% of the vitamin C in the solution remained intact during the 48 h incubation period. Furthermore, these findings showed that after 48 h of incubation for all of the Fe(II)-containing salts, the inclusion of vitamin C in the dispersions led to about 100% iron(II) in the solution. It is interesting to note that, for each set of mixed salts, the soluble iron content plateaued at approximately 3.2 mM as a function of the *x*-value, or the solubility limit of iron in these salts, during the experiments. To anticipate the extent of vitamin C oxidation during longer incubation times, future investigations can consider increasing the incubation temperature to as high as 50 °C.

In conclusion, ferrous pyrophosphate (Fe(II)PP) and mixed divalent metal Fe(II)-containing pyrophosphate salts with the general formula M_2(1–*x*)_Fe_2*x*_P_2_O_7_ (where 0 < *x* < 1 and M = Ca, Zn, or Mn) are synthesized and characterized in this work as possible essential mineral delivery systems. Additionally, following a 2 h incubation period at room temperature, we examine for the first time the dissolution behavior of these salts as a function of pH. Results show that Fe(II)PP was very poorly soluble at pH 3 (1.09 mM) and in the food-relevant pH (<0.5%), while it dissolved well at pH < 3 up to 11 mM (≈40%) at pH 1. Moreover, in the intermediate pH range of 4–7, all of the mixed salts exhibited very little iron dissolution (<0.5 mM), but in the gastric and intestinal pH ranges, they showed increased dissolved iron concentrations, up to 5 and 1.3 mM, respectively. Under gastric-relevant conditions (incubation at pH 2 and 37 °C for 75 min), only M = Ca (2.1 times greater than Fe(II)PP) exhibited improved soluble iron. Furthermore, the reactivity of these salts is shown in a black tea solution, which serves as a typical model system for phenolics, or catechins. The findings indicate that the black tea solution (ΔE* = 10.9) showed an acceptable color change for Fe(II)PP, while the salts with *x* = 0.470 when M = Zn and *x* = 0.086 when M = Mn show the least amount of discoloration (ΔE* = 8.6 and 6.7, respectively). Exploring the oxidation of vitamin C in the presence of these salts showed that over time (48 h), in the presence of Fe(II)PP 96% of vitamin C remains intact. Furthermore, the presence of the mixed salts lowered the oxidation extent of vitamin C down to 1.4 times when compared to Fe(II)PP (≈99% intact vitamin C). Our findings show that, when compared to vitamin C’s autoxidation in pure water under the same conditions, the mixed divalent metal Fe(II)-containing salts (i.e., *x* = 0.06, 0.086, and 0.053 where M = Ca, Zn, and Mn, respectively) have comparatively lower iron contents, making them potential dual-fortificants that do not enhance the oxidation of vitamin C. These results imply that the mixed Fe(II)-containing pyrophosphate salts and pure Fe(II)PP make potential candidates for multimineral fortification of foods high in vitamin C, with the primary objective being increased levels of intact vitamin C and soluble iron that are readily absorbed by the human body. Findings of this study indicate that the mixed divalent metal Fe(II)-containing pyrophosphate salts are potential delivery systems for dual-fortification of food products with necessary essential minerals. In future works, the pH-dependent dissolution behavior of iron from these mixed Fe(II)-containing pyrophosphate salts should be determined in realistic product formats. The stability of the fortified food product should be assessed depending on the level of fortification. If the concentration of any of the minerals per serving portion is close to or higher than the tolerable upper intake level (UL, i.e., the highest level of daily nutrient intake that is likely to pose no risk of adverse health effects), any potential toxicity should be assessed. After the required safety clearance, the mineral bioavailability should be measured as part of a diet. Finally, the oxidation of vitamin C in its presence in real food products and over real storage times is of interest and should be further investigated.

## Supporting Information Available

Preparation of the pure divalent metal pyrophosphate salts (Preparation S1); preparation of the mixed divalent metal pyrophosphate salts (Preparation S2); quantification of colors of the pyrophosphate salts (Figure S1); characterization method transmission electron microscopy and energy-dispersive X-ray spectroscopy (TEM–EDX) (Characterization S1); characterization method high-angle annular dark-field scanning TEM (HAADF–STEM) (Characterization S2); characterization method inductively coupled plasma atomic emission spectroscopy (ICP-AES) (Characterization S3); characterization method X-ray diffraction (XRD) spectroscopy (Characterization S4); characterization method Fourier transform infrared (FT-IR) spectroscopy (Characterization S5); dissolution method pH-dependent dissolution behavior of the pure and mixed divalent metal Fe(II)-containing pyrophosphate salts (Dissolution S1); dissolution behavior of iron from the pure Fe(II)PP and mixed divalent metal Fe(II)-containing pyrophosphate salts in gastric-mimicked conditions (Dissolution S2); color difference, Δ*E** (Figure S2); morphology of the salts by electron microscopy (Figures S3 and S4); characterization of the salts by XRD and FT-IR (Figure S5); interference of the presence of Ca, Zn, or Mn ion with the quantification of total iron in the ferrozine-based colorimetric assay (Figure S6); verification of the ferrozine assay with ICP-AES method (Figure S7); and effect of time on the oxidation of vitamin C in the presence of the pure and mixed divalent metal Fe(II)-containing pyrophosphate salts (Figure S8) (PDF)

## Abbreviations

Ca: calcium
CaPP: calcium pyrophosphate
Fe(II): ferrous iron
Fe(III): ferric iron
Fe(III)PP: iron(III) pyrophosphate
Fe(II)PP: iron(II) pyrophosphate
FT-IR: Fourier transform infrared spectroscopy
HAADF–STEM: high-angle annular dark-field scanning transmission electron microscopy
ICP-AES: inductively coupled plasma atomic emission spectroscopy
Mn: manganese
MnPP: manganese(II) pyrophosphate
TEM–EDX: transmission electron microscopy coupled to energy-dispersive X-ray spectroscopy
XRD: X-ray diffractometry
Zn: zinc
ZnPP: zinc pyrophosphate
