# Supplementary material for: Ferrous Pyrophosphate and Mixed Divalent Pyrophosphates as Delivery Systems for Essential Minerals
Source: ACS Food Sci Technol. 2024 Jun 5;4(6):1388–401. doi: 10.1021/acsfoodscitech.4c00050 (PMC11197097; doi:10.1021/acsfoodscitech.4c00050)
Supplement: Supplementary file 1 — fs4c00050_si_001.pdf [file fs4c00050_si_001.pdf]

Supporting Information

Ferrous Pyrophosphate and Mixed Divalent Pyrophosphates as Delivery Systems for Essential Minerals

Neshat Moslehi[a,1], Michiel van Eekelen[a], Krassimir P. Velikov[b,c,d], and Willem K. Kegel[a,*]

[a] Van 't Hoff Laboratory for Physical and Colloid Chemistry, Debye Institute for Nanomaterials Science, Utrecht University, Padualaan 8, 3584 CH Utrecht, The Netherlands

[b] Unilever Innovation Centre Wageningen, Bronland 14, 6708 WH, Wageningen, The Netherlands

[c] Soft Condensed Matter, Debye Institute for Nanomaterials Science, Utrecht University, Princetonplein 5, 3584 CC, Utrecht, The Netherlands

[d] Institute of Physics, University of Amsterdam, Science Park 904, 1098 XH, Amsterdam, The Netherlands

[*]Corresponding author: Email address W.K.Kegel@uu.nl, Tel: +31302532873

Email addresses authors:

Neshat Moslehi: n.moslehi@uu.nl

Michiel van Eekelen: m.vaneekelen@students.uu.nl

Krassimir P. Velikov: krassimir.velikov@unilever.com

Willem Kegel: w.k.kegel@uu.nl

---

[1] The current affiliation of the author: Laboratory of Self-Organizing Soft Matter, Department of Chemical Engineering and Chemistry, Eindhoven University of Technology, Eindhoven, The Netherlands

Supplementary material for "Ferrous Pyrophosphate and Mixed Divalent Pyrophosphates as Delivery Systems for Essential Minerals" by Moslehi, van Eekelen, Velikov, and Kegel

Table of Contents

1. Preparation methods....4

1.1. Preparation S1: Pure divalent metal pyrophosphate salts....4

1.2. Preparation S2: Mixed divalent metal pyrophosphate salts....4

2. Quantification of colors of the pyrophosphate salts....6

3. Characterization Methods....7

3.1. Characterization S1: Transmission Electron Microscopy and Energy-Dispersive X-ray spectroscopy (TEM-EDX)....7

3.2. Characterization S2: High-Angle Annular Dark Field Scanning TEM (HAADF-STEM)....7

3.3. Characterization S3: Inductively Coupled Plasma Atomic Emission Spectroscopy (ICP-AES)....8

3.4. Characterization S4: X-Ray Diffraction (XRD) Spectroscopy....8

3.5. Characterization S5: Fourier Transform Infrared (FT-IR) Spectroscopy....8

4. Dissolution Methods....9

4.1. Dissolution S1: pH-dependent dissolution behavior of the pure and mixed divalent metal Fe(II)-containing pyrophosphate salts....9

5. 4.1.1 Iron concentration measurement by a ferrozine-based colorimetric assay....9

6. 4.1.2. Elements concentration measurement by inductively coupled plasma – atomic emission spectroscopy (ICP-AES)....10

6.1. Dissolution S2: Dissolution behavior of iron from the pure Fe(II)PP and mixed divalent metal Fe(II)-containing pyrophosphate salts in gastric-mimicked conditions ..10

7. Color difference (Δ*E) 11**

8. Morphology of the salts by electron microscopy 12

9. Characterization of the salts by XRD and FT-IR 14

10. Interference of presence of Ca, Zn, or Mn ion with the quantification of total iron in the ferrozine-based colorimetric assay 19

11. Verification of the ferrozine assay with ICP-AES method.....................................19

12. Effect of time on the oxidation of vitamin C in the presence of the pure and mixed divalent metal Fe(II)-containing pyrophosphate salts 20

References 21

Supplementary material for "Ferrous Pyrophosphate and Mixed Divalent Pyrophosphates as Delivery Systems for Essential Minerals" by Moslehi, van Eekelen, Velikov, and Kegel

1. Preparation methods

1.1. Preparation S1: Pure divalent metal pyrophosphate salts

Pure divalent metal pyrophosphate salts were synthesized as references for comparative purposes. The preparation was done via a well-established co-precipitation method which has been described elsewhere previously [1–3]. Firstly, solutions of 1.286 mmol $FeSO_4.7H_2O$, $CaCl_2$, $ZnCl_2$, and $MnCl_2$ in 50 ml of MQ water were prepared separately. Following this, the solutions were added quickly (within 5 seconds) to a solution of 0.643 mmol $Na_4P_2O_7.10H_2O$ (NaPP) in 100 ml of MQ water to prepare iron (II) pyrophosphate ($Fe_2P_2O_7$, Fe(II)PP), calcium pyrophosphate ($Ca_2P_2O_7$, CaPP), zinc pyrophosphate ($Zn_2P_2O_7$, ZnPP), and manganese (II) pyrophosphate ($Mn_2P_2O_7$, MnPP), respectively. This was done while the NaPP solution was stirring vigorously (~ 400 rpm) with a magnetic stir bar. In the case of the Fe(II)PP a turbid light green, and in other cases turbid white/off-white dispersions were formed within a few seconds after the addition. The samples were then centrifuged at 3273 × *g* for 30 minutes in 50 ml volume polypropylene conical centrifuge tubes using an Allegra X-12R Centrifuge (Beckman Coulter, Brea, CA, USA). This was followed by washing the precipitate with MQ water twice. Finally, the salts were dried overnight in an oven at 45 °C (Fe(II)PP: 88%, CaPP: 74%, ZnPP: 80%, and MnPP: 72% yield).

1.2. Preparation S2: Mixed divalent metal pyrophosphate salts

The mixed divalent metal salts were prepared by the same procedure as the pure salts, by addition of 50 ml of a mixed solution of $FeSO_4.7H_2O$ plus $CaCl_2$, $ZnCl_2$, or $MnCl_2$ in MQ water to an aqueous solution of NaPP with a fixed concentration of pyrophosphate ions (6.43 mM, 100 ml). Three different series of mixed Fe(II)-containing pyrophosphate salts were prepared; each with a different second divalent metal (M) and different M and Fe(II) contents, based on the general formula $M_{2(1-x)}Fe_{2x}P_2O_7$ ($0 < x < 1$), for theoretical x-values

(0.05, 0.10, 0.25, and 0.50, coded as MMix1-4, where M = Ca, Zn, or Mn). After adding the mixed solution to NaPP, the solutions were stirred vigorously (~ 400 rpm) with a magnetic stir bar (final concentration of NaPP: 4.29 mM). In all ratios, a turbid dispersion was formed a few seconds after the addition. The samples were then centrifuged, washed, and dried in an oven following the same procedure as explained for the pure divalent metal salts. Consequently, the molar ratios were calculated based on which the x-value was found in the structural formula. The molar ratio of total metal ions ([M] + [Fe], final concentration: 8.573 mM) to pyrophosphate ions was 2:1. The average yields of the prepared mixed salts with M = Ca, Zn, and Mn were 33 ± 3%, 69 ± 10%, and 82 ± 7%, respectively.

2. Quantification of colors of the pyrophosphate salts

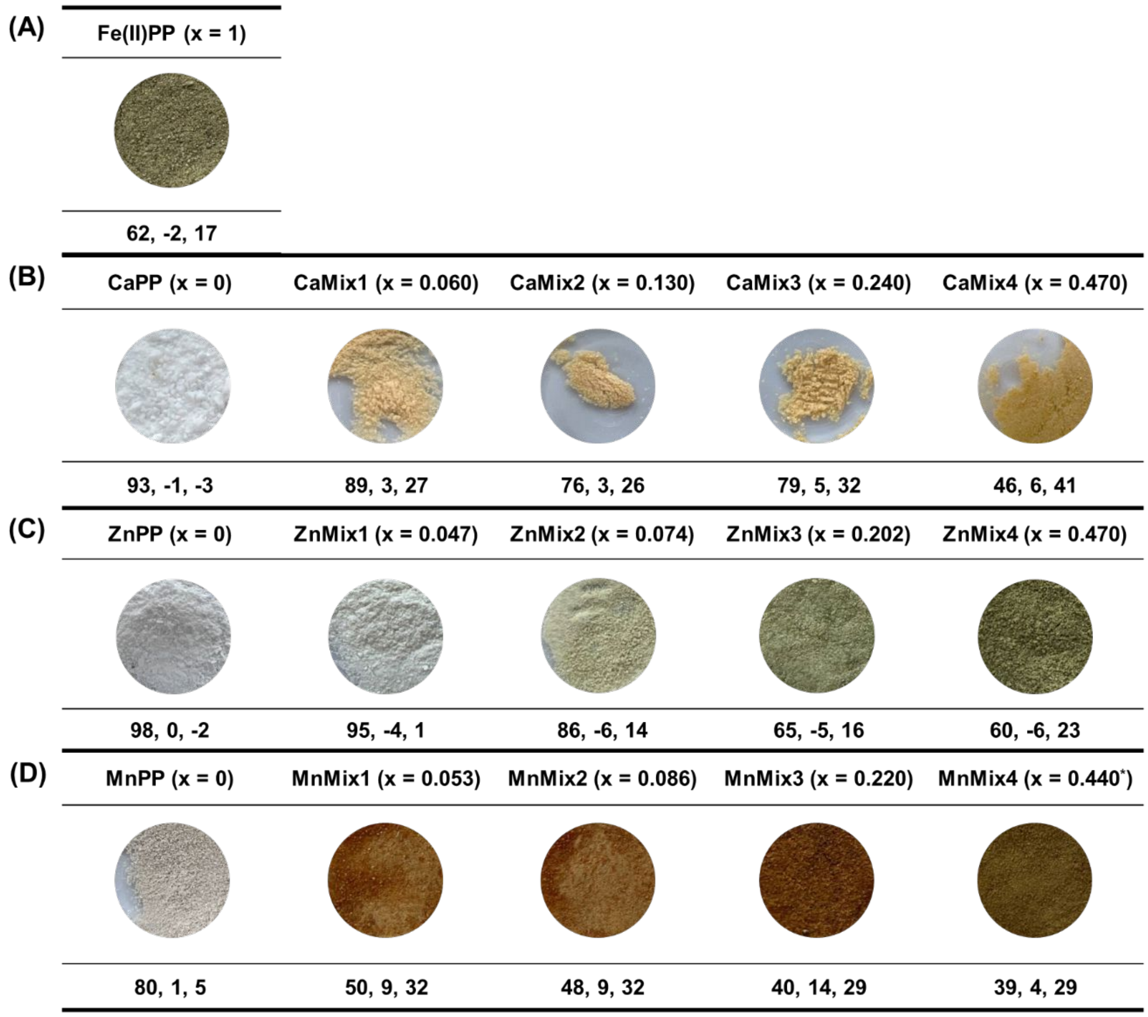

Figure S1. The images and details of color conversion (i.e., $L^*a^*b^*$ values) of (A) pure Fe(II)PP, the mixed pyrophosphate salts with the general formula $M_{2(1-x)}Fe_{2x}P_2O_7$ where (B) M = Ca, (C) M = Zn, and (D) M = Mn. The pure CaPP, ZnPP, and MnPP are shown for comparison. The details of color conversions of the salts are obtained by an online color measurement tool (https://imagecolorpicker.com/) and are reported under the images of the salts by L^*, a^*, b^* values from left to right, respectively.

*The average of the two x-values corresponding to the two morphological phases of the salt MnMix4 is used here (0.440).

3. Characterization Methods

3.1. Characterization S1: Transmission Electron Microscopy and Energy-Dispersive X-ray spectroscopy (TEM-EDX)

Water dispersions of the salts were dried on carbon-coated copper (for M = Ca and Zn) or nickel (for M = Zn) grids and analyzed by transmission electron microscopy and energy-dispersive X-ray spectroscopy (TEM-EDX). This was performed on a Talos™ F200X (Thermo Fisher Scientific, San Jose, CA, USA) operated at 200 kV. The elemental composition of the mixed salts was obtained from EDX and used for finding the experimental x-value based on the general formula of the mixed salts. The ratios of the atomic percentages (i.e., M/Fe = 2(1-x)/2x, M/P = 2(1-x)/2, Fe/P = 2x/2) were used to find x in the structural formula $M_{2(1-x)}Fe_{2x}P_2O_7$. The average x-values were incorporated in the general formula of the mixed divalent metal pyrophosphate salts to obtain the actual chemical formula of the salts. Elemental composition of the mixed salts in which M = Mn could not be obtained accurately by this technique (TEM-EDX) due to the overlapping Mn and Fe lines in the energy spectrum.

3.2. Characterization S2: High-Angle Annular Dark Field Scanning TEM (HAADF-STEM)

High-angle annular dark-field scanning TEM (HAADF-STEM) was performed on a Talos™ F200X (Thermo Fisher Scientific, San Jose, CA, USA) operated at 200 kV. The elemental mapping was recorded by assigning a color to each element. Color indications are as follows: second divalent metal (i.e., M = Ca, Zn, or Mn): green, iron: red, and phosphorus: blue.

3.3. Characterization S3: Inductively Coupled Plasma Atomic Emission Spectroscopy (ICP-AES)

For the mixed salts in which M = Mn, the chemical composition was obtained by elemental analysis using ICP-AES. After ensuring the identical morphology and homogeneous distribution of Fe and Mn in the salts (i.e., MnMix1-3), their samples were dissolved in 10 ml of a 2% HNO_3 solution to achieve optimal measurement concentration ranges. ICP-AES measurements were performed using an Optima 8300 instrument (PerkinElmer,Waltham, MA, USA) and in triplicate. Finally, the ratios of Mn/Fe and P/Fe were used to obtain the average x-value for each salt based on the general formula. For the salt with heterogeneous morphology (i.e., MnMix4), the approximate x-values were estimated using EDX analysis on the different morphological phases separately, as described in **Characterization S1**.

3.4. Characterization S4: X-Ray Diffraction (XRD) Spectroscopy

The dried powders of the salts were analyzed at room temperature with an AXS D2 Phaser powder X-ray diffractometer (Bruker®, Billerica, MA, USA), which was equipped with a LYNXEYE® detector in Bragg-Brentano mode. The radiation used was cobalt $K\alpha_{1,2}$, $\lambda = 1.79026$ Å, operated at 30 kV, 10 mA for $2\theta = 5$ to 70 degrees. A silicon holder was used, and the measurements were repeated twice on the salts from independent synthesis batches.

3.5. Characterization S5: Fourier Transform Infrared (FT-IR) Spectroscopy

FT-IR measurements were performed on dried powders of the salts using the KBr pellet technique [4] and by an FT-IR spectrometer (PerkinElmer, Waltham, MA, USA). 2.5 mg of the powder of each salt was mixed thoroughly with 250 mg of KBr powder (FT-IR grade), and dried in an oven at 60 °C overnight. Pellets were prepared using a press and the measurements were done in independent duplicate. The interferograms were collected over

the spectral range of 1600 – 400 cm^{-1} using a nominal resolution of 4 cm^{-1}, with a background scan recorded before each measurement.

4. Dissolution Methods

4.1. Dissolution S1: pH-dependent dissolution behavior of the pure and mixed divalent metal Fe(II)-containing pyrophosphate salts

The dried powders of the salts were re-dispersed in MQ water by stirring (~ 250 rpm) with a magnetic stir bar (final concentration: 10 mg/ml). Then, the pH of the dispersion was adjusted using a pH-stat device (Metrohm, Herisau, Switzerland) by the addition of 0.1 M HCl or 0.1 M NaOH. Subsequently, all dispersions were incubated at 1000 rpm using an Eppendorf ThermomixerR F1.5 (Eppendorf, Hamburg, Germany) at pH values ranging from one to eleven (steps of two pH units), for 2 h at 23 °C. After incubation, the final pH of each sample was measured. Finally, the samples were centrifuged at 15000 × *g* for 10 min using an Eppendorf Centrifuge 5415R and the supernatants were separated to quantify the dissolved elements concentrations.

5. 4.1.1 Iron concentration measurement by a ferrozine-based colorimetric assay

The concentration of the dissolved iron from Fe(II)PP and the mixed Fe(II)-containing pyrophosphate salts was monitored by a ferrozine-based colorimetric assay [5]. An excess amount of ascorbic acid (50 µl, 100 mM) was added to 50 μl sample (supernatant). After 30 minutes incubation of the sample with ascorbic acid, ferrozine (50 μl, 10 mM) was added. The absorbance at 565 nm was measured at room temperature by a SpectraMax M2e (Molecular Devices, Sunnyvale, CA, USA). Quantification of the dissolved iron was performed based on intensity and a calibration curve of $FeSO_4$ (0.0078 – 1 mM, $R^2 > 0.99$). Evaluation of the significance of differences in iron concentration was carried out by statistical analysis (significant at $p < 0.05$).

6. 4.1.2. Elements concentration measurement by inductively coupled plasma – atomic emission spectroscopy (ICP-AES)

Inductively coupled plasma–atomic emission spectroscopy (ICP-AES) was used for independent determination of iron concentration, to be compared to the ferrozine assay, as well as determination of other metals (Ca, Zn, and Mn) and phosphorus (P) concentrations in the salts. After incubation, the samples (supernatants) were 10 times diluted in 0.14 M HNO_3, before injection in the ICP-AES system (Agilent 5110 VDV; Agilent Technologies, Tokyo, Japan). All measurements were done in duplicate. The concentrations of iron, calcium, zinc, manganese, and phosphorus salts were determined using scandium as an internal standard. The limit of detection (LOD) values of iron, calcium, zinc, manganese, and phosphorus were 5, 20, 5, 1, and 50 μg/l, respectively.

6.1. Dissolution S2: Dissolution behavior of iron from the pure Fe(II)PP and mixed divalent metal Fe(II)-containing pyrophosphate salts in gastric-mimicked conditions

To get a primary indication of the bio-accessibility of iron and the minerals in the designed salts, dissolution of the salts in gastric-mimicked conditions was studied. The procedure was like the pH-dependent dissolution behavior except for the incubation conditions. The pH of the dispersions of the pure and mixed pyrophosphate in MQ water (final concentration: 10 mg/ml) was adjusted to pH 2 using a SevenExcellence Multiparameter pH meter (Mettler Toledo, Columbus, OH, USA) by the addition of 0.5M HCl. Subsequently, all dispersions were incubated at 37 °C for 75 min with 1000 rpm. After the incubation, the final pH of each sample was measured. Finally, the samples were centrifuged at 15000 × *g* for 10 minutes and the supernatants were isolated to quantify the dissolved Fe and the other elements (Ca, Zn, or Mn and P) concentrations (by ICP-AES). All measurements were performed in duplicate. To

quantify both Fe(II) and Fe(III) concentrations, the ferrozine-based colorimetric assay was done both with and without addition of excess amount of ascorbic acid. The presence of ascorbic acid results in reduction of Fe(III) to Fe(II) and consequently total amount of Fe in solution can be measured. In the case of addition of no ascorbic acid, the concentration of Fe(II) can be quantified directly. Accordingly, Fe(III) concentration is calculated by the difference between total Fe and Fe(II) concentrations.

7. Color difference (ΔE^*)

In this procedure, the $L^*a^*b^*$ value was taken at three different spots on the images of the samples. The absolute value of color difference, ΔE^*, corresponds to the distance between two points (blank and sample) within the $L^*a^*b^*$ color space and is calculated according to **Equation 1** [6,7].

$$\Delta E^* = [(\Delta L^*)^2 + (\Delta a^*)^2 + (\Delta b^*)^2]^{1/2} \qquad \textbf{(1)}$$

Where ΔL^*, Δa^*, Δb^* are the difference in the color space values before (blank) and after exposure to the mixed pyrophosphate salts (sample), respectively, **Figure S2**.

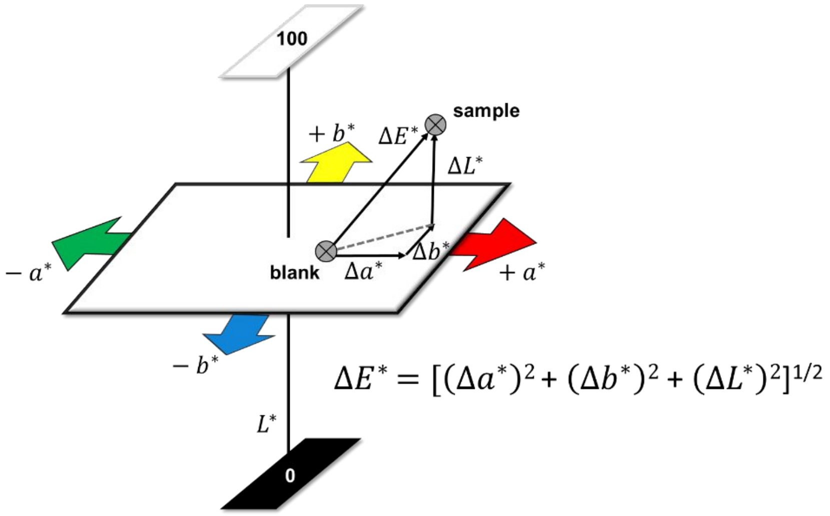

Figure S2. Schematic presentation and the equation of the color difference ΔE^* between two points (blank and sample) in the $L^*a^*b^*$ color space.

8. Morphology of the salts by electron microscopy

The morphology of the mixed divalent and the pure salts was investigated by electron microscopy, **Figure S3**.

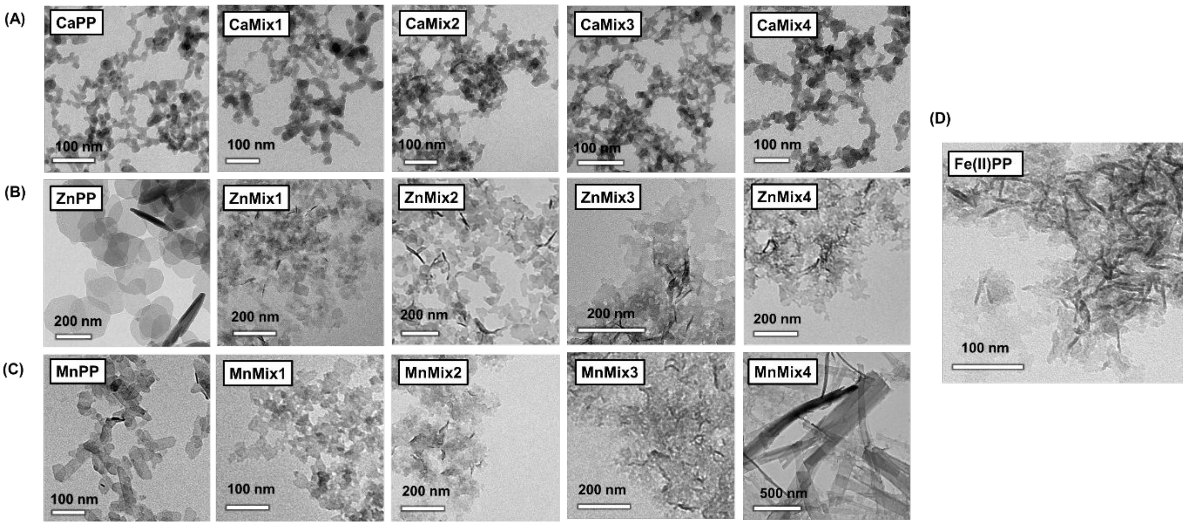

Figure S3. Morphology of the mixed divalent metal Fe(II)-containing pyrophosphate salts with the general formula $M_{2(1-x)}Fe_{2x}P_2O_7$ ($0 < x < 1$), coded as MMix1-4 where (A) M = Ca, (B) M = Zn, and (C) M = Mn obtained from TEM imaging. The morphology of the pure salts; (D) Fe(II)PP (x = 1), and other pure divalent metal pyrophosphate salts; CaPP, ZnPP, and MnPP (x = 0 for M = Ca, Zn, and Mn, respectively) are shown for comparison as well. Comparison indicates that all the mixed Fe(II)-containing salts yield uniform morphology and homogeneous particles, except for the salt MnMix4 which shows segregation into two coexisting structural phases.

The dried dispersions of these salts showed diverse morphologies. The CaPP yielded interconnected aggregates of noticeably small (10-20 nm) spherical particles, **Fig. S3A**, which was in line with a previous study [1]. Moreover, the TEM image showed that ZnPP formed relatively larger (roughly 200 nm) platelets of 10-25 nm thick, whereas MnPP was a mixture of round and rod-shaped platelets of maximum 100 nm long, see **Figure S3B-C**. In addition, The TEM images of Fe(II)PP showed large aggregates of 50-100 nm irregularly-shaped particles, **Figure S3D**. TEM images showed that inclusion of Ca as the second divalent metal (i.e., M = Ca) in the Fe(II)-containing pyrophosphate salts resulted in

homogeneous and similar morphologies to CaPP for all the designed x-values, indicating aggregates of small (10-20 nm) round particles for the salts CaMix1-4, **Figure S3A**. In the case of M = Zn, the mixed salts yielded aggregates of thin platelets of approximately 100-200 nm large, **Figure S3B**. Detailed analysis of the results obtained from TEM-EDX and ICP-AES measurements on the mixed pyrophosphate salts in which M = Mn (i.e., MMix1-4) suggested that incorporating Mn as the second divalent metal alongside Fe(II) in the pyrophosphate matrix yields formation of a uniform morphology which was observed to be irregularly-shaped particles of 100-200 nm in all the mixed salts (x-values), except for the salt MnMix4 (i.e., the mixed salt with the highest iron content), **Figure S3C.** Finally, the morphology of the Fe(II)PP was assessed by electron microscopy, **Figure S3B**. The TEM imaging of the dried dispersions of this salt showed formation of micron-sized aggregates with homogeneous distribution of Fe and P throughout the salt matrix.

The elemental distribution in the mixed divalent metal Fe(II)-containing pyrophosphate salts was visualized by HAADF-STEM. Homogenous distribution of the elements M (= Ca, Zn, or Mn), Fe, and P was observed by elemental mapping of the mixed salts CaMix1-4, **Figure S4A**, ZnMix1-4, **Figure S4B**, and MnMix1-3, **Figure S4C**. In line with the results of TEM-EDX, the elemental mapping of the salt MnMix4 showed different distribution of Fe for Fe-rich and Mn-rich (i.e., Fe-poor) coexisting morphologies.

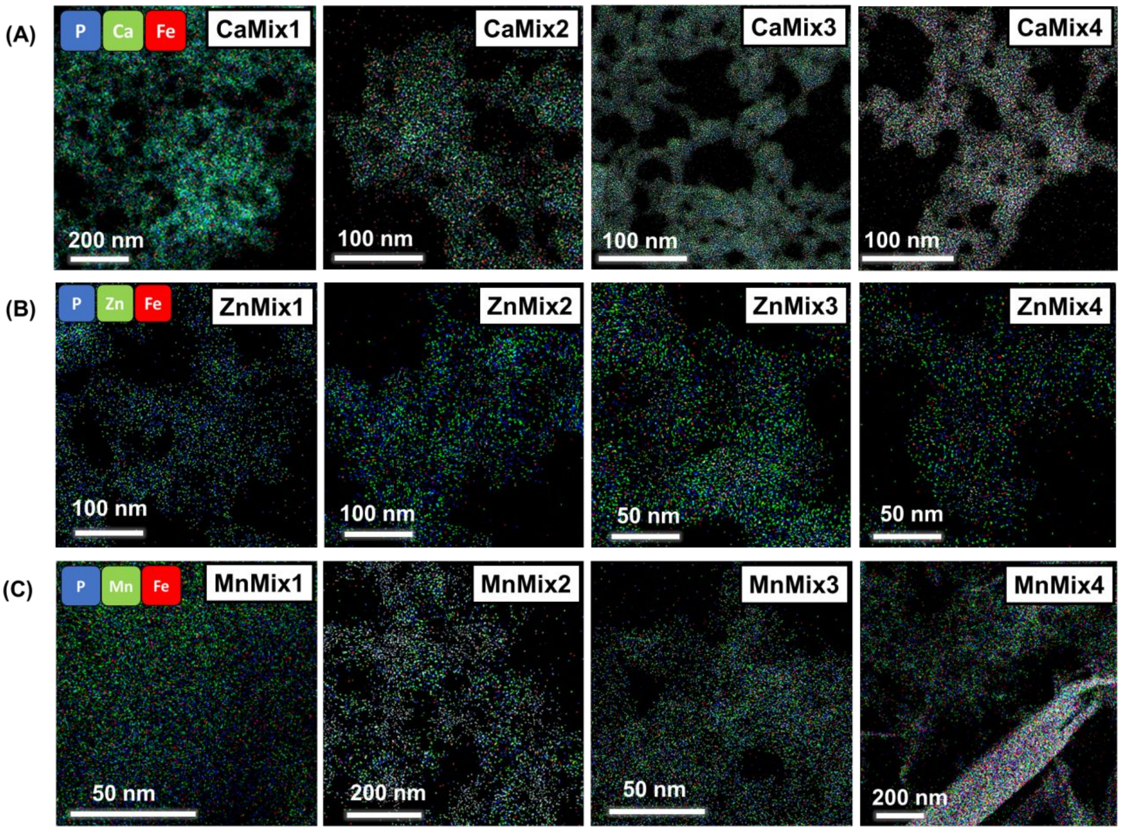

Figure S4. Elemental mapping performed by HAADF-STEM on the mixed Fe(II)-containing pyrophosphate salts with the general formula $M_{2(1-x)}Fe_{2x}P_2O_7$, coded as MMix1-4 where (A) M = Ca, (B) M = Zn, and (C) M = Mn. Color indications are: second divalent metal (M = Ca, Zn, or Mn): green; iron (Fe): red; and phosphorous (P): blue. Elemental mapping clearly shows homogeneous distribution of the elements in all the mixed salts except for the salt MnMix4.

9. Characterization of the salts by XRD and FT-IR

It has previously been shown that the crystallinity of metal pyrophosphate particles can depend on the valence match/mismatch of the metal and pyrophosphate ions [1]. In general, divalent metals are expected to form crystalline compounds with pyrophosphate ions due to the less complicated stoichiometric ratio to reach neutrality [1]. This was confirmed for the pure salts CaPP, ZnPP, and MnPP by XRD diffractograms and FT-IR spectra, **Figure S5**. The X-ray diffraction spectra for CaPP and ZnPP indicated clear signals of highly crystalline structures which are similar to the previously reported diffractograms for monoclinic calcium

pyrophosphate tetrahydrate β (m-CPPT β) [8] and polymorphs of zinc pyrophosphate [9,10], **Figure S5A-B** (left), respectively. In XRD diffractogram of MnPP, we observed only one signal of crystallinity at approximately 2θ = 8 degree which has not been reported before [11–13], **Figure S5C** (left). This could be explained by the formation of different crystalline structures resulting from the fast co-precipitation method compared to the solid-state preparation. In addition, the synthesis method proposed in the current work is similar to synthesis of amorphous MnPP which has been reported before [13]. Finally, the diffractogram of Fe(II)PP, which is shown for comparison in **Figure S5**, exhibited only noise and a broad peak indicating amorphous structure for this salt, despite the less complicated stoichiometry of Fe^{2+} and $P_2O_7^{4-}$ ions for reaching neutrality [2,14].

Supplementary material for "Ferrous Pyrophosphate and Mixed Divalent Pyrophosphates as Delivery Systems for Essential Minerals" by Moslehi, van Eekelen, Velikov, and Kegel

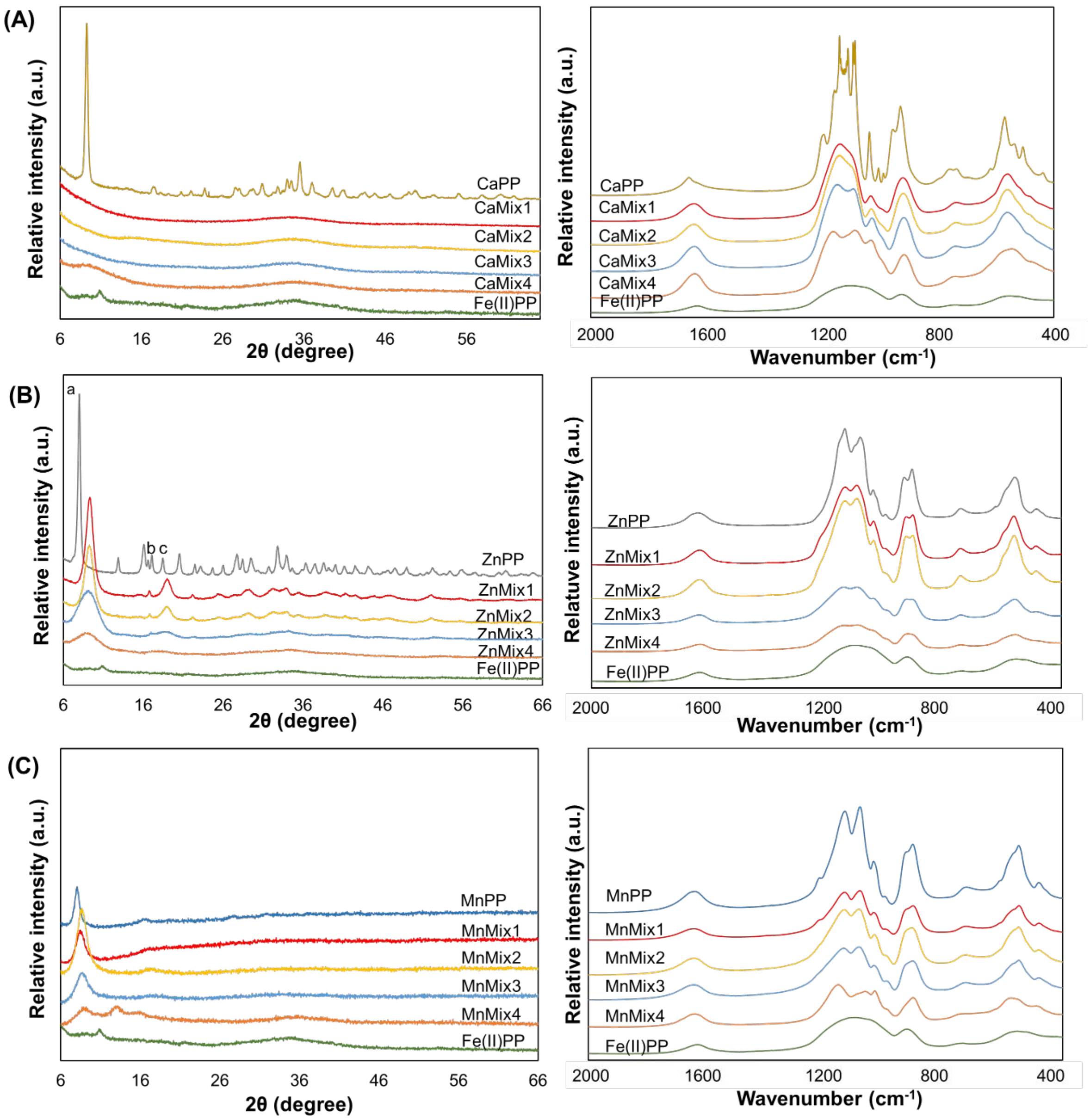

Figure S5. XRD diffractograms (left) and FT-IR spectra (right) of the mixed Fe(II)-containing pyrophosphate salts with the general formula $M_{2(1-x)}Fe_{2x}P_2O_7$ ($0 < x < 1$) where (A) M = Ca, (B) M = Zn, and (C) M = Mn. The XRD and FT-IR of the pure CaPP, ZnPP or MnPP and Fe(II)PP are shown for comparison as well. (A) The salts CaMix1-4 show broad peaks in their XRD and FT-IR spectra which is a sign of their amorphous structures. (B) A gradual transformation in the crystalline structure was observed in the XRD and FTIR spectra of the salts ZnMix1-4 upon increasing Fe content. Eventually the characteristic peak at 9.5 degree for crystalline structure and the chemical bond vibration peaks in the FT-IR spectra of the mixed salts ZnMix3 and ZnMix4 appeared broad and smooth. (C) The characteristic peak in the XRD spectra of MnPP appeared

broader upon increasing Fe content in the salts MnMix1-4. For the same reason, the peaks correspond to thir chemical bonds in the pyrophosphate ions appeared broad and smooth.

The crystallinity of the mixed divalent metal Fe(II)-containing pyrophosphate salts was investigated by X-ray powder diffraction analysis, **Figure S5** (left). The XRD diffractograms of the mixed salts were not similar to any of the existing XRD reference patterns in the international center for diffraction data (ICDD, https://www.icdd.com). Comparing the XRD patterns of the the pure CaPP and Fe(II)PP to their mixed salts (i.e., CaMix1-4) showed that inclusion of Ca alongside Fe(II) in the pyrophosphate matrix results in amorphous structures of these mixed salts and therefore appearing noise and broad peaks in their XRD diffractograms, **Figure S5A** (left).

However, in the case of Zn as the second divalent metal (M) comparison between the XRD spectra of the mixed salts (i.e., ZnMix1-4) and the pure ZnPP and Fe(II)PP specified a gradual transformation of crystalline structure upon increasing iron content in these samples, **Figure S5B** (left). The characteristic peak at $2\theta = 8$ degree (a), which appeared sharp and strong for ZnPP, was shifted to approximately 9.5 degree in the mixed salts. Moreover, the intensity and sharpness of this peak decreased noticeably upon increasing iron content in the mixed salts. Additionally, the peaks at 17 degree (b), 19 degree (c), and in the range of 25–35 degrees appeared broader with a decreased intensity in the salts ZnMix1-3 compared to ZnPP. Ultimately, these peaks disappeared in the spectra of the salt ZnMix4.

The XRD patterns of the mixed Fe(II)-containing pyrophosphate salts with M = Mn (i.e., MnMix1-4) were consistent with the pattern of MnPP, **Figure S5C** (left). The characteristic peak at approximately $2\theta = 8$ degree appeared in all the mixed salts and broadened upon increasing iron content in these salts. Eventually, the multiple peaks appearing for the salt

MnMix4 can be likely due to the multiple conformational phases resulting from the physical segregation observed in the morphology of this salt, **Figure S5C**.

The details of chemical bonding in the pure salts Fe(II)PP, CaPP, ZnPP, and MnPP were investigated by FT-IR spectrometry, **Figure S5** (right). The FT-IR spectrum for CaPP, ZnPP, and MnPP showed sharp and well-defined bands, whereas the peaks appeared broad and smooth for Fe(II)PP at the same wavenumbers. The sharper and well-defined bands were expected for crystalline materials. Spectral broadening in Fe(II)PP was a result of its amorphous nature [1,15]. Despite different broadness, peak positions of the main characteristic peaks matched with each other because the pyrophosphate groups are the main vibrationally active species. The characteristic peaks which appeared around 500-600 cm^{-1} are attributed to bending for O-P-O bonds in the P_2O_7 groups. The signals observed at 745 and 945 cm^{-1} are assigned to symmetric and asymmetric vibrations in P-O-P, respectively. The peaks in the range of 1000 to 1200 cm^{-1} correspond to P-O stretching vibration frequencies. All the peaks observed for the $P_2O_7^{4-}$ anion are similar to previous reports [3,10,11,15–17].

The details of chemical bonds in the structure of the mixed Fe(II)-containing pyrophosphate salts were explored by FT-IR spectroscopy **Figure S5** (right). The analysis of the FT-IR spectra of the mixed salts showed that peak positions coincided between the salts because the vibrations only correlate with the chemical bonds in the pyrophosphate ions. Pyrophosphate vibrations showed sharp and well-defined peaks for the crystalline salts, whereas the same peaks appeared broad and smooth for the amorphous salts. The peak positions matched with the wavenumbers observed for pyrophosphate group in the pure salts; Fe(II)PP, CaPP, ZnPP, and MnPP.

10. Interference of presence of Ca, Zn, or Mn ion with the quantification of total iron in the ferrozine-based colorimetric assay

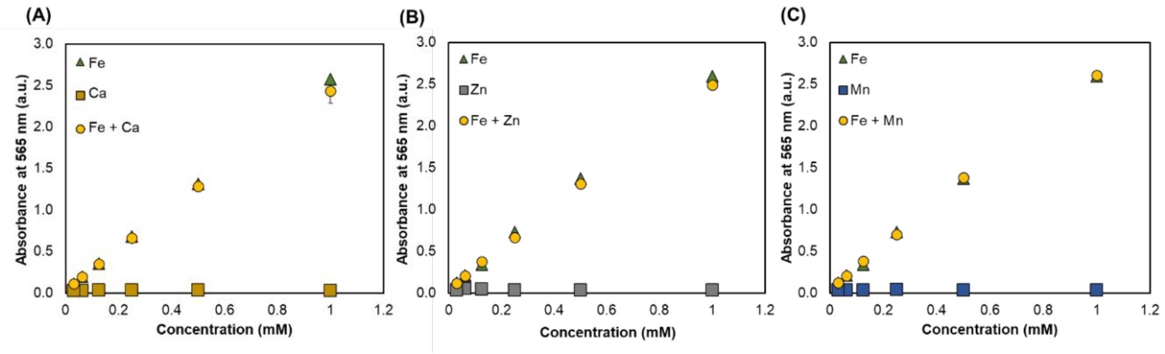

Figure S6. Interference of presence of Ca, Zn, or Mn ion with the quantification of total iron in the ferrozine-based colorimetric assay. Absorbance of the ferrozine complex at 565 nm in the presence of increasing concentration of Fe, second divalent metal (M), and a mixture containing their equimolar concentrations where (A) M = Ca, (B) M = Zn, and (C) M = Mn. Presence of none of the studied divalent metals (i.e., Ca, Zn, and Mn) resulted in

absorbance or interference with the absorbance of the Fe-ferrozine complex at 565 nm.

11. Verification of the ferrozine assay with ICP-AES method

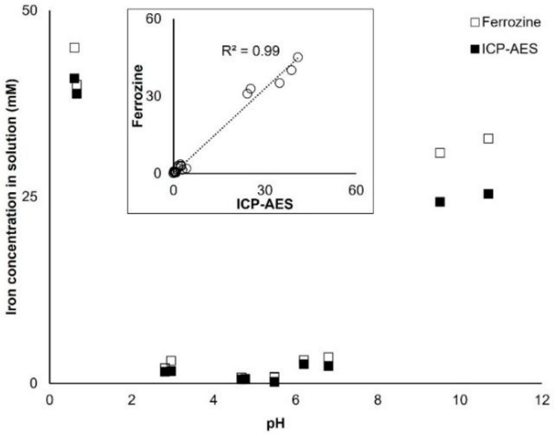

Figure S7. Verification of the ferrozine assay with ICP-AES method. Dissolution of iron from Fe(II)PP as a function of pH determined by ICP-AES (filled markers) and by the ferrozine (open markers) methods. The inset depicts the correlation between the iron concentration measured using both methods (including the experiments at gastric-mimicked conditions). The iron concentrations in solution measured by the two different methods are found to be in good agreement ($R^2 = 0.99$).

12. Effect of time on the oxidation of vitamin C in the presence of the pure and mixed divalent metal Fe(II)-containing pyrophosphate salts

To examine the oxidation of vitamin C in the presence of the pure and mixed divalent metal pyrophosphate salts in longer times, generation of DHA was studied (and normalized to the concentration of the generated DHA in the absence of any salts) as a function of time (1 h, 2 h, and 48 h incubation) at room temperature (23 °C).

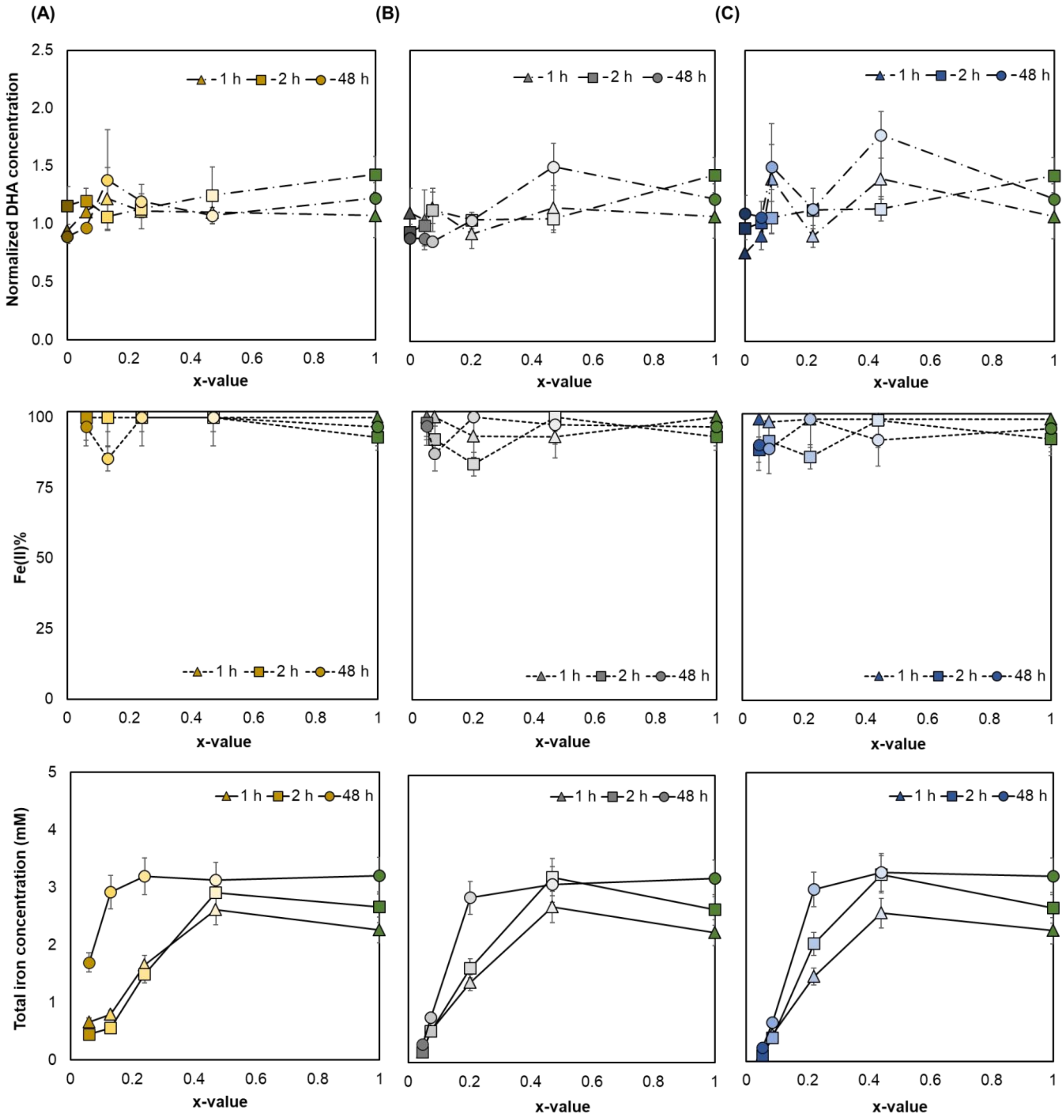

Figure S8. The effect of time on the oxidation of vitamin C in the presence of Fe(II)PP and the mixed salts.

Normalized DHA concentration (dash dotted lines, top), the percentage of the dissolved iron (II) (dashed lines, middle) and the total dissolved iron concentration (solid lines, bottom) from the $M_{2(1-x)}Fe_{2x}P_2O_7$ ($0 \leq x \leq 1$) salts where (A) M = Ca, (B) M = Zn, and (C) M = Mn after 1 h, 2 h, and 48 h at 23 °C. The results indicate that over 48 h, 96% of the total vitamin C in the solution remained intact.

Monitoring the normalized DHA concentration in the case of Fe(II)PP showed no significant difference and fluctuated in the range of 1.1 - 1.5 over time, x = 1 in **Figure S8** (top). Moreover, results indicated that although this value was noisy in the case of the mixed (vs. x-values) over time, the DHA concentration was always < 2 times higher for all the mixed salts, compared to their absence, **Figure S8A-C** (top). After 48 h incubation, the maximum value of DHA (among all mixed salts) was measured for the mixed salts with x = 0.130, 0.470, and 0.440 when M = Ca, Zn, and Mn, respectively. This means that over the 48 h incubation, approximately 96% of the total vitamin C present in the solution remained intact.

References

(1) van Leeuwen, Y. M.; Velikov, K. P.; Kegel, W. K. Morphology of Colloidal Metal Pyrophosphate Salts. *RSC Adv* **2012**, *2* (6), 2534–2540. https://doi.org/10.1039/c2ra00449f.

(2) Rossi, L.; Velikov, K. P.; Philipse, A. P. Colloidal Iron(III) Pyrophosphate Particles. *Food Chem* **2014**, *151*, 243–247. https://doi.org/10.1016/j.foodchem.2013.11.050.

(3) Moslehi, N.; Bijlsma, J.; de Bruijn, W. J. C.; Velikov, K. P.; Vincken, J.-P.; Kegel, W. K. Design and Characterization of Ca-Fe(III) Pyrophosphate Salts with Tunable PH-Dependent Solubility for Dual-Fortification of Foods. *J Funct Foods* **2022**, *92*, 105066. https://doi.org/10.1016/J.JFF.2022.105066.

(4) Drenchev, N. L.; Chakarova, K. K.; Lagunov, O. V.; Mihaylov, M. Y.; Ivanova, E. Z.; Strauss, I.; Hadjiivanov, K. I. In Situ FTIR Spectroscopy as a Tool for Investigation of Gas/Solid Interaction: Water-Enhanced CO_2 Adsorption in UiO-66 Metal-Organic Framework. *JoVE (Journal of Visualized Experiments)* **2020**, *2020* (156), e60285. https://doi.org/10.3791/60285.

(5) Stookey, L. L. Ferrozine: A New Spectrophotometric Reagent for Iron. *Anal Chem* **1970**, *42* (7), 779–781. https://doi.org//10.1021/ac60289a016.

(6) Araki, H.; Kim, J.; Zhang, S.; Banks, A.; Crawford, K. E.; Sheng, X.; Gutruf, P.; Shi, Y.; Pielak, R. M.; Rogers, J. A. Materials and Device Designs for an Epidermal UV Colorimetric Dosimeter with Near Field Communication Capabilities. *Adv Funct Mater* **2017**, *27* (2), 1604465. https://doi.org/10.1002/ADFM.201604465.

(7) Poynton, C. Digital Video and HD. *Digital Video and HD* **2012**. https://doi.org/10.1016/C2010-0-68987-5.

(8) Gras, P.; Rey, C.; Marsan, O.; Sarda, S.; Combes, C. Synthesis and Characterisation of Hydrated Calcium Pyrophosphate Phases of Biological Interest. *Eur J Inorg Chem* **2013**, *2013* (34), 5886–5895. https://doi.org/10.1002/ejic.201300955.

(9) Karaphun, A.; Sawadsitang, S.; Duangchuen, T.; Chirawatkul, P.; Putjuso, T.; Kumnorkaew, P.; Maensiri, S.; Swatsitang, E. Influence of Calcination Temperature on Structural, Morphological, and Electrochemical Properties of $Zn_2P_2O_7$ Nanostructure. *Surfaces and Interfaces* **2021**, *23*, 100961. https://doi.org/10.1016/J.SURFIN.2021.100961.

(10) Petrova, M. A.; Shitova, V. I.; Mikirticheva, G. A.; Popova, V. F.; Malshikov, A. E. New Data on $Zn_2P_2O_7$ Phase Transformations. *J Solid State Chem* **1995**, *119* (2), 219–223. https://doi.org/10.1016/0022-4596(95)80035-N.

(11) Brouzi, K.; Ennaciri, A.; Harcharras, M. THERMAL TRANSFORMATIONS AND INFRARED STUDIES OF $Mn_2P_2O_7 \cdot 2H_2O$. *http://dx.doi.org/10.1080/10426500490468137* **2010**, *179* (7), 1329–1339. https://doi.org/10.1080/10426500490468137.

(12) Boonchom, B.; Baitahe, R. Synthesis and Characterization of Nanocrystalline Manganese Pyrophosphate $Mn_2P_2O_7$. *Mater Lett* **2009**, *63* (26), 2218–2220. https://doi.org/10.1016/J.MATLET.2009.07.028.

(13) Schneider, S.; Collin, R. L. Crystal Structure of Manganese Pyrophosphate Dihydrate, $Mn_2P_2O_7 \cdot 2H_2O$. *Inorg Chem* **1973**, *12* (9), 2136–2139. https://doi.org/10.1021/IC50127A040/ASSET/IC50127A040.FP.PNG_V03.

(14) Lai, Y.; Liang, X.; Yin, G.; Yang, S.; Wang, J.; Zhu, H.; Yu, H. Infrared Spectra of Iron Phosphate Glasses with Gadolinium Oxide. *J Mol Struct* **2011**, *1004* (1–3), 188–192. https://doi.org/10.1016/J.MOLSTRUC.2011.08.003.

(15) Singh, R. K.; Srivastava, M.; Prasad, N. K.; Awasthi, S.; Dhayalan, A.; Kannan, S. Iron Doped β-Tricalcium Phosphate: Synthesis, Characterization, Hyperthermia Effect, Biocompatibility and Mechanical Evaluation. *Materials Science and Engineering C* **2017**, *78*, 715–726. https://doi.org/10.1016/j.msec.2017.04.130.

(16) Zheng, J. C.; Ou, X.; Zhang, B.; Shen, C.; Zhang, J. F.; Ming, L.; Han, Y. D. Effects of Ni^{2+} Doping on the Performances of Lithium Iron Pyrophosphate Cathode Material. *J Power Sources* **2014**, *268*, 96–105. https://doi.org/10.1016/j.jpowsour.2014.05.147.

(17) Kosova, N. V.; Rezepova, D. O.; Podgornova, O. A.; Slobodyuk, A. B.; Petrov, S. A.; Avdeev, M. A Comparative Study of Structure, Air Sensitivity and Electrochemistry of Sodium Iron Pyrophosphates $Na_{2-x}Fe_{1+x/2}P_2O_7$ (x = 0; 0.44). *Electrochim Acta* **2017**, *235*, 42–55. https://doi.org/10.1016/j.electacta.2017.03.058.
